# Population-based blood screening for preclinical Alzheimer’s disease in a British birth cohort at age 70

**DOI:** 10.1093/brain/awaa403

**Published:** 2021-01-22

**Authors:** Ashvini Keshavan, Josef Pannee, Thomas K Karikari, Juan Lantero Rodriguez, Nicholas J Ashton, Jennifer M Nicholas, David M Cash, William Coath, Christopher A Lane, Thomas D Parker, Kirsty Lu, Sarah M Buchanan, Sarah E Keuss, Sarah-Naomi James, Heidi Murray-Smith, Andrew Wong, Anna Barnes, John C Dickson, Amanda Heslegrave, Erik Portelius, Marcus Richards, Nick C Fox, Henrik Zetterberg, Kaj Blennow, Jonathan M Schott

**Affiliations:** 1 Dementia Research Centre, UCL Queen Square Institute of Neurology, University College London, London, UK; 2 Clinical Neurochemistry Laboratory, Department of Psychiatry and Neurochemistry, Institute of Neuroscience and Physiology, The Sahlgrenska Academy at University of Gothenburg, Sahlgrenska University Hospital, Mölndal, Sweden; 3 Wallenberg Centre for Molecular and Translational Medicine, University of Gothenburg, Gothenburg, Sweden; 4 Department of Old Age Psychiatry, Institute of Psychiatry, Psychology and Neuroscience, King’s College London, London, UK; 5 National Institute for Health Research Biomedical Research Centre for Mental Health and Biomedical Research Unit for Dementia at South London and Maudsley NHS Foundation Trust, London, UK; 6 Department of Medical Statistics, London School of Hygiene and Tropical Medicine, University of London, London, UK; 7 MRC Unit for Lifelong Health and Ageing at UCL, London, UK; 8 Institute of Nuclear Medicine, University College London Hospitals NHS Foundation Trust, London, UK; 9 UK Dementia Research Institute Fluid Biomarkers Laboratory, UK DRI at UCL, London, UK

**Keywords:** Alzheimer’s disease, amyloid imaging, dementia, beta-amyloid, tau, epidemiology

## Abstract

Alzheimer’s disease has a preclinical stage when cerebral amyloid-β deposition occurs before symptoms emerge, and when amyloid-β-targeted therapies may have maximum benefits. Existing amyloid-β status measurement techniques, including amyloid PET and CSF testing, are difficult to deploy at scale, so blood biomarkers are increasingly considered for screening. We compared three different blood-based techniques—liquid chromatography-mass spectrometry measures of plasma amyloid-β, and single molecule array (Simoa) measures of plasma amyloid-β and phospho-tau181—to detect cortical ^18^F-florbetapir amyloid PET positivity (defined as a standardized uptake value ratio of >0.61 between a predefined cortical region of interest and eroded subcortical white matter) in dementia-free members of Insight 46, a substudy of the population-based British 1946 birth cohort. We used logistic regression models with blood biomarkers as predictors of amyloid PET status, with or without age, sex and *APOE* ε4 carrier status as covariates. We generated receiver operating characteristics curves and quantified areas under the curves to compare the concordance of the different blood tests with amyloid PET. We determined blood test cut-off points using Youden’s index, then estimated numbers needed to screen to obtain 100 amyloid PET-positive individuals. Of the 502 individuals assessed, 441 dementia-free individuals with complete data were included; 82 (18.6%) were amyloid PET-positive. The area under the curve for amyloid PET status using a base model comprising age, sex and *APOE* ε4 carrier status was 0.695 (95% confidence interval: 0.628–0.762). The two best-performing Simoa plasma biomarkers were amyloid-β_42/40_ (0.620; 0.548–0.691) and phospho-tau181 (0.707; 0.646–0.768), but neither outperformed the base model. Mass spectrometry plasma measures performed significantly better than any other measure (amyloid-β_1-42/1-40_: 0.817; 0.770–0.864 and amyloid-β composite: 0.820; 0.775–0.866). At a cut-off point of 0.095, mass spectrometry measures of amyloid-β_1-42/1-40_ detected amyloid PET positivity with 86.6% sensitivity and 71.9% specificity. Without screening, to obtain 100 PET-positive individuals from a population with similar amyloid PET positivity prevalence to Insight 46, 543 PET scans would need to be performed. Screening using age, sex and *APOE* ε4 status would require 940 individuals, of whom 266 would proceed to scan. Using mass spectrometry amyloid-β_1-42/1-40_ alone would reduce these numbers to 623 individuals and 243 individuals, respectively. Across a theoretical range of amyloid PET positivity prevalence of 10–50%, mass spectrometry measures of amyloid-β_1-42/1-40_ would consistently reduce the numbers proceeding to scans, with greater cost savings demonstrated at lower prevalence.

## Teaser

Keshavan *et al.* show that plasma Aβ_1-42/1-40_ and a plasma Aβ composite measured by liquid chromatography–mass spectrometry perform better than various blood biomarkers measured by single molecular array, and better than age, sex and *APOE* ε4, in screening for preclinical Alzheimer’s disease in the British 1946 birth cohort.

## Introduction

A core early feature of Alzheimer’s disease is brain deposition of amyloid-β, which is detectable *in vivo* using amyloid PET ligands binding fibrillar amyloid-β (Klunk *et al.*, 2004; Morris *et al.*, 2016), and by CSF testing showing reduced concentrations of amyloid-β_42_ (Motter *et al.*, 1995; Olsson *et al.*, 2016) or amyloid-β_42_/amyloid-β_40_ (Slaets *et al.*, 2013; Toledo *et al.*, 2013; Pannee *et al.*, 2016). These methods now have consensus appropriate use criteria (Johnson *et al.*, 2013; Shaw *et al.*, 2018), are incorporated into current clinical guidelines (National Institute for Health and Care Excellence, 2018) and research criteria for Alzheimer’s disease diagnosis (Dubois *et al.*, 2014; Jack *et al.*, 2018), and are increasingly used in clinical trials to either confirm Alzheimer’s disease pathology in symptomatic individuals, or to identify asymptomatic individuals at risk, who are now defined as having preclinical Alzheimer’s disease. However, amyloid PET is expensive, limited in accessibility and involves ionizing radiation. CSF sampling is relatively invasive, confers risk to individuals with coagulopathies, and requires suitably trained personnel. As neither method is likely to be viable for population-based screening, several blood-based approaches have been developed.

A meta-analysis of studies published until 2015 (Olsson *et al.*, 2016) yielded conflicting results on the ability of plasma amyloid-β to distinguish Alzheimer’s disease dementia from controls, or mild cognitive impairment (MCI) progressing to Alzheimer’s disease dementia from stable MCI. These mixed findings were related particularly to heterogeneity of comparisons; older studies compared clinically diagnosed Alzheimer’s disease cases with various non-pathologically defined control groups, while newer studies compared amyloid-positive and -negative groups as defined by PET or CSF. Initially, methods used to quantify plasma amyloid-β also lacked sensitivity, but from 2016 onward, highly sensitive immunoassays and high-throughput mass spectrometry methods were developed for measuring plasma amyloid-β. Several studies, utilizing either of these methods, probed the ability of blood-based assays to distinguish individuals with *in vivo* gold standard biomarker evidence of Alzheimer pathology (i.e. either cortical amyloid PET uptake or lowered CSF amyloid-β_1-42_ or amyloid-β_1-42_/amyloid-β_1-40_) from controls without evidence of Alzheimer pathology. Some publications focused on memory clinic populations (mixed cohorts of cognitively healthy elderly subjects, MCI and dementia), such as the Japanese National Center for Geriatrics and Gerontology, the Australian Imaging, Biomarker and Lifestyle Study of Ageing (Nakamura *et al.*, 2018), the Swedish BioFINDER study (Palmqvist *et al.*, 2018), and the Washington University cohort (Schindler *et al.*, 2019). Some studies specifically prospectively investigated individuals reporting subjective cognitive decline, such as the Amsterdam Dementia Cohort (Verberk *et al.*, 2018), and Insight pre-AD (Vergallo *et al.*, 2019). Taken together, in memory clinic populations, plasma amyloid-β_42_/amyloid-β_40_ was able to predict concurrent PET or CSF amyloid status with sensitivities of 70–76% and specificities of 72–78% (accuracy varied from 68% to 97% across cohorts measured by different assays). Plasma amyloid-β_42_/amyloid-β_40_ measured by an immunoprecipitation–mass spectrometry (IP-MS) method was also able to predict conversion from PET-negative to PET-positive status more than 1.5 years later, with individuals who had plasma amyloid-β_42_/amyloid-β_40_ < 0.1218 being 15 times more likely to convert from PET-negative to PET-positive than those above this cut-off point (Schindler *et al.*, 2019).

More recently, plasma phospho-tau181 (p-tau181) has emerged as a potential biomarker of amyloid-β positivity, with studies reporting an accuracy of 76–88% for predicting amyloid PET status (Mielke *et al.*, 2018; Karikari *et al.*, 2020). Plasma p-tau181 may have some additive predictive value with plasma amyloid-β_42/40_ measured by immunoassays (Janelidze *et al.*, 2020). Validation of these approaches has largely focused on mixed cohorts (of individuals who are cognitively normal, those with MCI and those with dementia).

Methods for identifying amyloid-β-positive individuals at scale will take priority if disease-modifying therapies are licensed. A key possible reason for the negative results seen in many of the earlier amyloid-lowering therapy trials was lack of biomarker evidence of Alzheimer’s disease in the full recruited study populations (Doody *et al.*, 2014; Salloway *et al.*, 2014; Vandenberghe *et al.*, 2016). More recent trials have therefore turned to ensuring that participants have Alzheimer’s pathology as defined by mutation carrier status in dominantly inherited disease or by amyloid biomarkers. Examining results of 4-year follow-up in the Dominantly Inherited Alzheimer’s Network Trial Unit (DIAN-TU) study of solanezumab and gantenerumab, where the presence of Alzheimer’s disease was defined by mutation carrier status, primary cognitive endpoints were not met (Bateman, 2020). In mild symptomatic sporadic Alzheimer’s disease, the ENGAGE trial of aducanumab also did not meet its primary end point but the similarly designed EMERGE trial did show benefit at 78 weeks (Biogen, 2019), which has led to a submission for regulatory approval to the US Food and Drug Administration. Among the key limitations of the amyloid-lowering trials in even mild symptomatic sporadic Alzheimer’s disease is the possibility that the window of opportunity for preventing cognitive decline in such individuals has already passed. With primary prevention trials such as the Anti-Amyloid Treatment in Asymptomatic Alzheimer disease (A4) Study of solanezumab (Sperling *et al.*, 2020) aiming to target those who may still be in that window, population screening for amyloid-β positivity will need to shift focus to asymptomatic individuals.

The main aim of our study was therefore to compare directly the ability of three blood-based candidates for detecting cerebral amyloid-β deposition—liquid chromatography-mass spectrometry (LC-MS) measures of plasma amyloid-β, and single molecule array (Simoa) measures of plasma amyloid-β and phospho-tau181—to determine amyloid PET status in a population-based sample of dementia-free individuals at age ∼70 years, drawn from the longest continuously participating British birth cohort study. In addition, we assessed correlations between biomarkers, probed associations of demographic factors with blood biomarkers, and examined the potential cost savings of deploying each screening method across a range of amyloid PET-positivity prevalence.

## Materials and methods

### Study design and participants

The Medical Research Council (MRC) National Survey for Health and Development (NSHD; the British 1946 birth cohort) followed an initial sample of 5362 individuals from their birth in mainland Britain during 1 week in March 1946, over 24 waves of data collection. Insight 46 is a prospective substudy of 502 members at age 69–71 years, undertaken at University College London (UCL); previous publications have detailed the eligibility criteria and substudy protocol (Lane *et al.*, 2017), and compared the characteristics of Insight 46 participants to those of the larger NSHD cohort (James *et al.*, 2018). Ethical approval for Insight 46 was given by the National Research Ethics Service Committee London (reference 14/LO/1173). All participants gave written informed consent according to the Declaration of Helsinki.

### Neuroimaging

Dynamic PET-MRI data were acquired simultaneously on a single Biograph mMR 3 T PET-MRI scanner, as described previously (Lane *et al.*, 2017). Amyloid-β burden was assessed by analysing the PET data acquired at a 10-min period, 50 min after intravenous injection of 370 MBq ^18^F-florbetapir. Global standardized uptake value ratio (SUVR) was calculated by normalizing the uptake in a predefined cortical region of interest (Landau *et al.*, 2013) to that in eroded subcortical white matter. Amyloid PET status was obtained by fitting a two-component Gaussian mixture model of SUVR in all participants with adequate PET data, and taking the 99th percentile of the lower (amyloid PET-negative) Gaussian as the cut-off point (SUVR = 0.61, equivalent to 17 centiloids). SUVR ≥ 0.61 was defined as PET positive and SUVR < 0.61 as PET negative. Although all study assessments were designed to be completed in a single day, 59 individuals (13.4% of those included in the analysis) had their PET scans on a different day to blood sampling due to PET tracer availability or scanner maintenance issues; the median delay between the blood test and the scan in these individuals was 0.131 years [interquartile range (IQR): 0.060–0.211 years].

Whole brain volume, subcortical white matter hyperintensity volume and total intracranial volume were extracted from the MRI data for use as covariates in models examining the associations of age with plasma amyloid-β biomarkers. Whole brain volume was extracted from T_1_-weighted images using the automated Brain Multi-Atlas Propagation and Segmentation (BMAPS) technique (Leung *et al.*, 2011). Subcortical white matter hyperintensity volume was extracted from T_1_ and FLAIR images using the automated Bayesian Model Selection (BaMoS) algorithm (Sudre *et al.*, 2015), which excludes infratentorial regions, followed by visual quality control to exclude individuals with white matter lesions characteristic of demyelination or large cortical infarcts inappropriately segmented as subcortical white matter hyperintensities. Total intracranial volume was extracted using Statistical Parametric Mapping software version 12 (Malone *et al.*, 2015).

### Cognitive assessment

Neuropsychological testing (Lane *et al.*, 2017; Lu *et al.*, 2019) included a summary measure of cognition [the Mini-Mental State Examination (MMSE); Folstein *et al.*, 1975], tests of episodic memory [Wechsler memory scale-revised logical memory test (Wechsler, 1987) and 12-item Face-Name Associative Memory Exam (FNAME-12); Papp *et al.*, 2014], and processing speed (Wechsler adult intelligence scale-revised digit symbol substitution test (DSS); Wechsler, 1981]. The preclinical Alzheimer’s cognitive composite (PACC) was derived as the sum of *z*-scores of the MMSE, delayed logical memory (LMD), FNAME-12 and DSS (Lu *et al.*, 2019). Participant and informant history of cognitive concerns and of prior neurological diagnoses was also elicited. Dementia was defined by expert consensus (informed by clinical history, informant history and MMSE score < 26). MCI was defined as participant or informant concern regarding participant’s cognition, and either LMD score ≥1.5 standard deviations (SD) below the mean, or DSS score ≥1.5 SD below the mean.

### Blood sampling and processing

Non-fasted venous blood was sampled between 09:30 and 11:00 h, using a tourniquet and 21G or 23G butterfly needle with a BD Vacutainer collecting system, into 8.5 ml gel separator serum tubes and 10 ml EDTA plasma tubes. Whole blood was transported and centrifuged at room temperature, at 2000*g* for 10 min, within 30 min of sampling. Plasma and serum supernatant aliquots of 0.5 ml were stored in polypropylene screw-top cryovials at −80°C within 60 min of sampling.

*APOE* ε4 genotyping of the single nucleotide polymorphisms rs439358 and rs7412 (Rawle *et al.*, 2018) was used to define *APOE* ε4 carrier status as carrier (one or two ε4 alleles) or non-carrier.

All blood assays were performed blinded to clinical information.

### Nomenclature for amyloid-β assays

Throughout this manuscript we refer to amyloid-β_42_ and amyloid-β_40_ where assays do not specify the starting amino acid of the relevant peptides, and to amyloid-β_1-42_ and amyloid-β_1-40_ where assays quantify the specific peptides starting at the first amino acid residue of the amyloid-β sequence.

### Simoa assays

#### Plasma amyloid-β_40_ and amyloid-β_42_

One 0.5 ml aliquot of plasma from each individual was thawed to room temperature over 1 h and vortexed for 2 s. Next, 0.3 ml was pipetted into a 1.5 ml polypropylene tube for centrifugation at 13 000*g* for 10 min; 0.1 ml of the supernatant was pipetted onto each of two plates, for analysing amyloid-β_40_ and amyloid-β_42_, respectively. Samples were analysed in duplicate, using the same batch of reagents (Simoa Aβ40 and Aβ42 kits), on the same HD-1 Analyser (Quanterix) at UCL. Results were accepted if the intra-assay coefficient of variation (CV) across the duplicates was <15%. Run validation controls prepared using stock peptide solutions from the kits indicated inter-assay CV < 30%.

#### Plasma p-tau181

Plasma p-tau181 was measured on the Simoa HD-1 instrument at the University of Gothenburg (Quanterix) using a recently published method that has been validated both analytically and clinically (Benussi *et al.*, 2020; Karikari *et al.*, 2020). Briefly, the assay used the p-tau181-specific monoclonal antibody AT270 for capture and the N-terminal mouse monoclonal antibody Tau12 that binds the N-terminal epitope 6-QEFEVMEDHAGT-18 on full-length human tau for detection. The calibrator was recombinant full-length recombinant tau-441 phosphorylated *in vitro* by glycogen synthase kinase 3β (#TO8-50FN, SignalChem). The specificity of the assay for tau forms that contain the indicated epitopes was previously demonstrated by mass spectrometry (Karikari *et al.*, 2020). All Insight 46 samples measured above the assay’s lower limit of quantification of 1.0 pg/ml. For further details, see the Supplementary material.

### LC-MS plasma amyloid-β assay

Extended methods are available in the Supplementary material. Calibrators were prepared using recombinant amyloid-β_1-38_, amyloid-β_1-40_ and amyloid-β_1-42_ (rPeptide) added to 8% bovine serum albumin in phosphate-buffered saline. Recombinant ‘heavy’ peptides (^15^N-uniformly labelled amyloid-β_1-38_, amyloid-β_1-40_ and amyloid-β_1-42_; rPeptide) were added to samples and calibrators prior to preparation and used as internal standards. Pooled plasma samples from the University of Gothenburg were used to track assay performance over different days, and showed inter-assay CV < 5%.

After a single thaw, amyloid-β peptides were immunoprecipitated from 0.25 ml of each sample, with anti-amyloid-β antibodies 4G8 (epitope 17–27 in the amyloid-β sequence) and 6E10 (epitope 1–16, both antibodies from BioLegend) coupled to Dynabeads™ M-280 Sheep Anti-Mouse IgG magnetic beads (Thermo Fisher Scientific), performed using a KingFisher™ Flex Purification System (Thermo Fisher Scientific). After immunoprecipitation, eluates in 0.1 ml of 0.5% formic acid were vacuum centrifuged and stored at −80°C.

Prior to liquid chromatography-tandem mass spectrometry (LC-MS/MS), the dried eluates were resuspended in 20% acetonitrile and 4% concentrated ammonia in water, and injected into the LC-MS system (a Dionex UltiMate LC system and a Thermo Scientific Q Exactive^TM^ Quadrupole-Orbitrap^TM^ hybrid mass spectrometer). Chromatographic separation was achieved using basic mobile phases and a reversed-phase monolith column at a flow rate of 0.3 ml/min. The mass spectrometer, operating in parallel reaction monitoring mode, was set to isolate the 4+ charge state precursors of the amyloid-β peptides. Product ions specific for each precursor (Supplementary Table 1) were selected and summed to calculate the chromatographic areas for each peptide and its corresponding internal standard. The area ratio of each analyte to its internal standard was used for quantification in samples and calibrators. Peptides analysed included amyloid-β_1-38_, amyloid-β_1-40_, amyloid-β_1-42_ and amyloid-β_−3-40_ (also known as APP669–711). An LC-MS composite was also generated by taking the average of the *z*-scores of the amyloid-β_−3-40_/amyloid-β_1-42_ and amyloid-β_1-40_/amyloid-β_1-42_ ratios, after the method of Nakamura *et al.* (2018).

### Statistical analysis

Analyses were performed in Stata Version 14.2 (Stata Corporation, Texas, USA).

#### Descriptive statistics

Mann-Whitney U-tests for non-normally distributed continuous variables, Student’s *t*-test for normally distributed continuous variables and χ^2^ tests for categorical variables were used to compare the amyloid PET-positive and PET-negative groups, for all who had a high-quality amyloid scan.

#### Inter-biomarker correlations

Correlations between blood biomarker values were assessed in all individuals for whom blood biomarker data were available. As blood biomarker values were positively skewed, logarithmic transformation was used before assessing Pearson correlations between assays.

#### Associations between blood biomarkers and demographics, accounting for imaging outcomes and cognition

For analyses of the associations of demographic factors with blood biomarkers, individuals were included if they had a full set of blood biomarker data, a high-quality amyloid PET scan, and were dementia-free. Figure 2 shows the process of inclusion and exclusion of participants to derive the dementia-free group. Although all individuals were born in the same week, age at the time of blood testing indicated time of attendance within the testing period of 2.6 years. Associations of blood biomarkers with age were tested using univariable linear regression, subsequently additionally adjusting for sex, *APOE* ε4 carrier status, SUVR, PACC, whole brain volume and subcortical white matter hyperintensity volume. Models including whole brain volume or subcortical white matter hyperintensity volume also adjusted for total intracranial volume. Unadjusted differences in blood biomarkers between the sexes were assessed by Mann-Whitney U-tests. For all these analyses, the outcome variables were log-transformed blood biomarkers or ratios, apart from the LC-MS composite which was examined without transformation.

Linear regression was used to investigate the associations of SUVR and *APOE* ε4 carrier status, adjusted for age and sex, with log-transformed blood biomarkers (or non-transformed LC-MS composite) as the dependent variable. Possible interactive effects of sex and SUVR, sex and *APOE* ε4 carrier status, and SUVR and *APOE* ε4 carrier status were investigated by including appropriate interaction terms. Model assumptions of linearity were checked by examination of residuals.

### Blood biomarker concordance with amyloid PET and relative costs of screening

**Figure 1:**
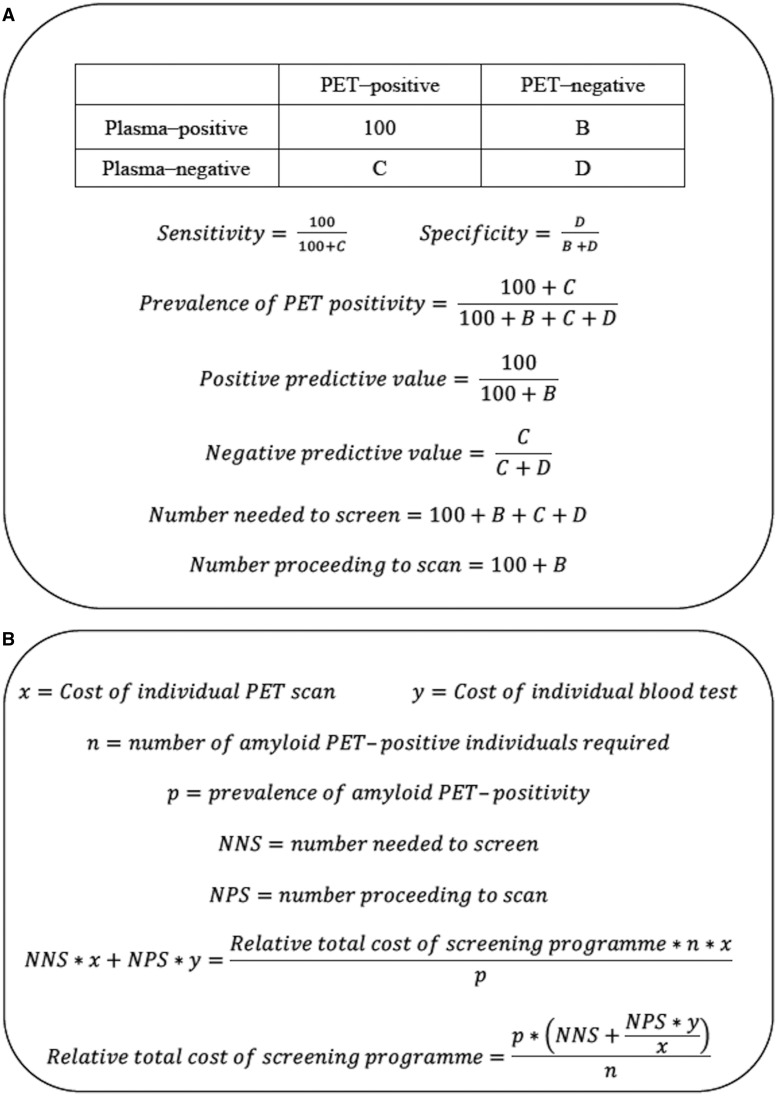
**Formulae.** (**A**) Calculations for number needed to screen to obtain 100 amyloid PET-positive individuals. Sensitivity and specificity are independent of prevalence, and are fixed once the cut-off point is chosen (e.g. at Youden’s index). If prevalence is specified, the first three simultaneous equations can be solved for *B*–*D*. The positive and negative predictive values of the plasma test and the number needed to screen can then be calculated by the following three equations. (**B**) Calculations for relative cost of the screening programme. These calculations are based on specified costs of an individual PET scan (*x*) and blood test (*y*), number needed to prescreen (NNS) with blood test and number proceeding to scan (NPS), to obtain a specified number of amyloid PET-positive individuals (*n*) in the context of a known estimated population prevalence of amyloid PET positivity (*p*). It is assumed that *x* and *y* include initial setup costs.

To assess the contribution of blood biomarkers to prediction of binary amyloid PET status, logistic regression models were constructed using blood biomarkers as predictors, with and without inclusion of age, sex and *APOE* ε4 carrier status. The model predictions were then used in receiver operating characteristics (ROC) analyses and the areas under the ROC curves (AUC) were compared using DeLong tests. The best model derived from each assay platform was defined as having the highest AUC. Plasma cut-off points were determined using Youden’s index as provided by the output of the ROC analysis. The resulting values of sensitivity and specificity were applied across a range of 10–50% theoretical prevalence of amyloid PET positivity to calculate the positive and negative predictive values of the blood test, the total number needed to screen by blood test, and number proceeding to PET scan, to identify 100 amyloid PET-positive individuals. These calculations were performed by solving for the contents of the 2 × 2 table generated in each case, using the equations shown in Fig. 1A. The relative costs of screening programmes using the different blood tests were examined by solving the equations in Fig. 1B, at different theoretical cost ratios of individual blood tests to PET scan, and across a range of 10–50% theoretical prevalence of amyloid PET positivity.

### Sensitivity analyses

**Figure 2:**
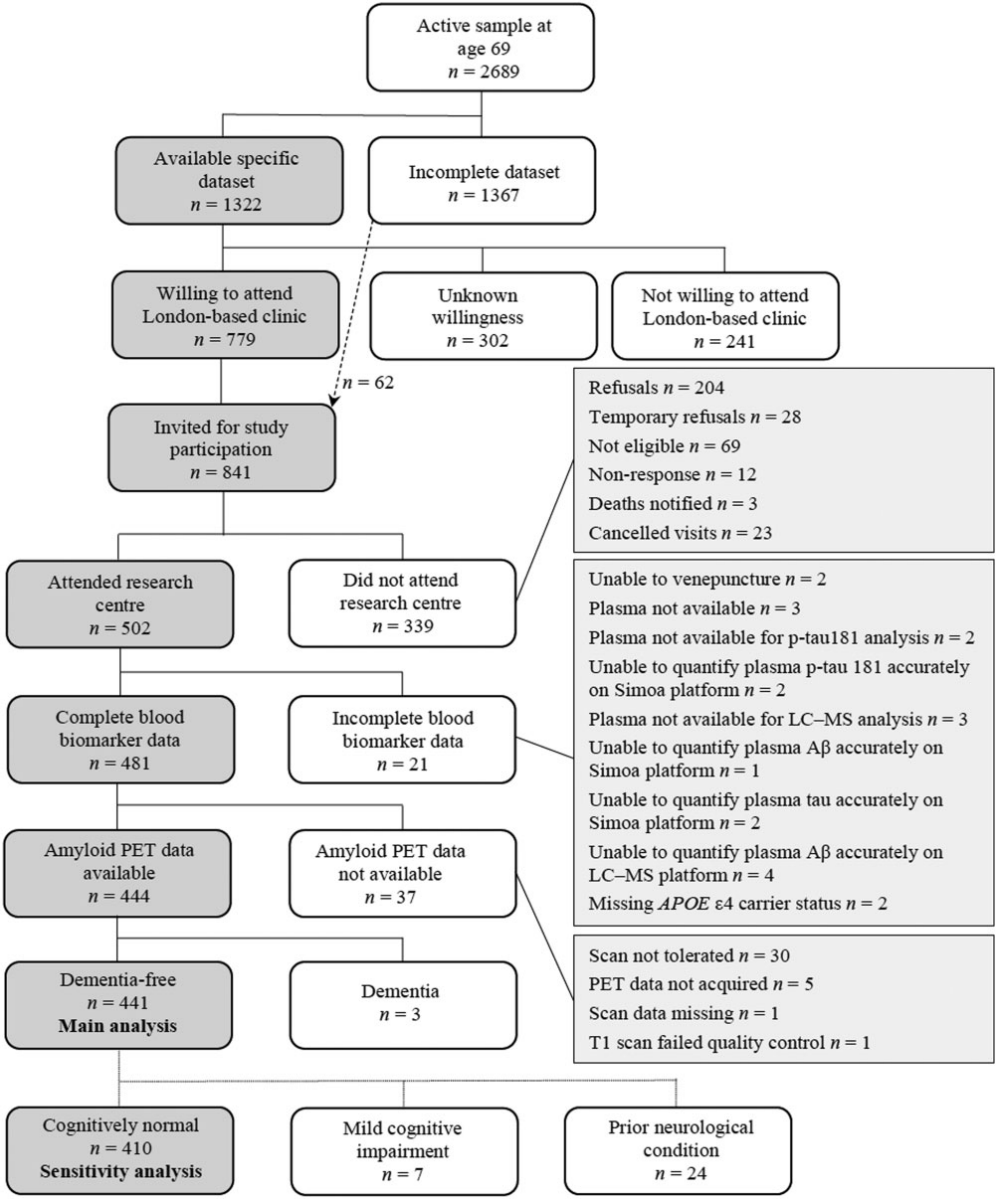
**Recruitment flow chart.** The chart shows an overview of Insight 46 recruitment from the MRC NSHD and summary of blood biomarker data available [modified with permission (James *et al.*, 2018) under the terms of the Creative Commons Attribution 4.0 International Licence (http://creativecommons.org/licenses/by/4.0/)]. The derivation of the dementia free group (used for the main analyses) and the cognitively normal group (used in the sensitivity analysis for prediction of amyloid PET status) is shown. Aβ = amyloid-β.

ROC analyses were performed for amyloid PET status within *APOE* ε4 subgroups (non-carrier versus carrier) and after further excluding those with prior neurological diagnoses and those who fulfilled criteria for MCI (Fig. 2). The contribution of time between blood test and scan, and of educational attainment, were also ascertained.

To examine whether the choice of amyloid PET cut-off point significantly affected the performance of the best-performing unadjusted plasma tests, sensitivity analyses were undertaken using a range of definitions of amyloid PET status either side of the cut-off point of 0.61 that had previously been determined by mixture modelling (SUVR cut-off points between 0.57 and 0.65), with recalculation of the Youden’s index plasma cut-off point in each case.

### Data availability

The data sharing policy is available on the NSHD Data Sharing website (see https://skylark.ucl.ac.uk/NSHD/doku.php).

## Results

### Participants: descriptive statistics

Of 502 participants assessed in Insight 46, 481 had a complete set of blood biomarker data, 444 had a high-quality amyloid PET scan, of whom 441 (92% of those with complete blood biomarker data) were dementia-free (Fig. 2). Table 1 summarizes the characteristics of all individuals included in the analysis; 82 of 441 individuals (18.6%) were amyloid PET positive. Compared with PET-negative individuals, PET-positive individuals had lower MMSE (median PET positive 29 versus PET negative 30, *P = *0.018) and PACC scores [mean (SD): −0.122 (0.726) versus 0.046 (0.668), *P *=* *0.044], were more likely to be *APOE* ε4 carriers (57.3% versus 22.0%, *P < *0.0001), and had higher whole brain volumes (median (IQR), ml: 1126 (1965–1187) versus 1096 (1026–1153), *P *=* *0.018). Supplementary Table 2 summarizes the characteristics of all dementia-free individuals, including those with missing data, and all those excluded from analysis.

**Table 1:** Characteristics of dementia-free participants with full blood biomarker and amyloid PET data

Characteristic	**Amyloid PET-negative** **(*n = *359)**	**Amyloid PET-positive** **(*n* = 82)**	*P*	**All dementia-free with amyloid scan** **(*n* = 441)**
Age at blood sampling, years	70.7 (0.7)	70.6 (0.6)	0.521	70.7 (0.7)
Sex, % female	49.6	54.9	0.367	50.6
*APOE* ε4 status, % carrier	22.0	57.3	**<0.0001**	28.6
MMSE	30 (29, 30)	29 (28, 30)	**0.018**	30 (29, 30)
PACC (z-score)	0.046 (0.668)	−0.122 (0.726)	0.044	0.015 (0.682)
Educational attainment by age 26, *n* (%)			0.571	
No qualification	52 (14.5)	13 (15.9)		65 (14.7)
Vocational	18 (5.0)	5 (6.1)		23 (5.2)
O-level/equivalent	91 (25.4)	21 (25.6)		112 (25.4)
A-level/equivalent	129 (35.9)	29 (35.4)		159 (35.8)
Degree/equivalent	69 (19.2)	14 (17.1)		83 (18.8)
*n* (%) individuals with blood sample and amyloid PET scan not done on same day	52 (14.5)	7 (8.5)	0.621	59 (13.4)
Time between blood sample and amyloid PET scan for individuals who did not have them on the same day, y	0.126 [0.063, 0.210] *n = *52	0.134 [0.038, 0.350] *n* = 7	0.672	0.131 [0.060, 0.211] *n = *59
Total intracranial volume, ml	1421 [1335, 1507]	1448 [1381, 1542]	0.076	1427 [1341, 1517]
Whole brain volume, ml	1096 [1026, 1153] *n = *357	1126 [1065, 1187]	**0.018**	1100 [1034, 1162] *n* = 439
WMHV, ml	2.8 [1.5, 6.6] *n = *348	3.5 [1.8, 7.0] *n = *79	0.329	3.1 [1.6, 6.8] *n* = 427
Serum creatinine, µmol/l	73 [63, 83]	73 [64, 87]	0.375	73 [63.5, 84]
Body mass index	27.4 [24.3, 30.4]	26.2 [24.1, 28.8]	0.054	27.3 [24.3, 30.2]
Simoa plasma Aβ_40_, pg/ml	289 [255, 319]	285 [257, 328]	0.601	288 [256, 322]
Simoa plasma Aβ_42_, pg/ml	19.9 [17.1, 22.6]	18.1 [15.5, 22.9]	**0.011**	19.6 [16.7, 22.7]
Simoa plasma Aβ_42_/Aβ_40_	0.068 [0.059, 0.078]	0.061 [0.052, 0.072]	**0.001**	0.066 [0.058, 0.077]
Simoa plasma p-tau181, pg/ml	8.5 [6.1, 12.2]	12.8 [9.2, 16.0]	**<0.0001**	9.2 [6.4, 12.9]
LC-MS plasma Aβ_1-38_, pg/ml	24.8 [21.6, 27.9]	24.5 [21.6, 28.7]	0.960	24.8 [21.6, 8.0]
LC-MS plasma Aβ_1-40_, pg/ml	284 [255, 314]	285 [260, 311]	0.976	284 [257, 314]
LC-MS plasma Aβ_1-42_, pg/ml	29.6 [24.9, 34.2]	23.7 [20.3, 27.2]	**<0.0001**	28.6 [23.4, 33.3]
LC-MS plasma Aβ_−3-40_, pg/ml	30.1 [24.4, 35.7]	29.9 [24.4, 34.9]	0.911	30.1 [24.4, 35.7]
LC-MS plasma Aβ_1-42_/Aβ_1-40_	0.104 [0.093, 0.116]	0.082 [0.075, 0.090]	**<0.0001**	0.099 [0.087, 0.113]
LC-MS plasma composite	−0.154 (0.736)	0.667 (0.718)	**<0.0001**	−0.001 (0.799)

Values are expressed as mean (SD) for normally distributed variables, median [interquartile range] for skewed variables, and percentages for binary and categorical variables. The significance of differences between amyloid PET*-*negative and PET*-*positive participants was determined by Mann-Whitney U-tests (for continuous variables excepting age and LC*-*MS plasma composite), *t-*tests (for age and LC*-*MS plasma composite), two sample tests of proportions (for binary variables) and Kruskal-Wallis equality of proportions rank test (for educational attainmen*t).* Aβ = amyloid-β; MMSE = Mini-Mental State Examination; NFL = neurofilament light chain; NSHD = National Survey of Health and Development; PACC = preclinical Alzheimer’s cognitive composite; p-tau181 = phospho-tau181; WMHV = white matter hyperintensity volume.

### Inter-biomarker correlations

Weak positive correlations were observed between Simoa ln amyloid-β_42_ and LC-MS ln amyloid-β_1-42_ (*r *=* *0.207, *P *=* *0.001), Simoa ln amyloid-β_40_ and LC-MS ln amyloid-β_1-40_ (*r *=* *0.406, *P < *0.0001), and Simoa ln amyloid-β_42_/amyloid-β_40_ and LC-MS ln amyloid-β_1-42_/amyloid-β_1-40_ (*r *=* *0.189, *P = *0.003). Simoa ln p-tau181 was weakly negatively correlated with LC-MS ln amyloid-β_1-42_ (*r* = −0.204, *P *=* *0.001) and LC-MS ln amyloid-β_1-42_/amyloid-β_1-40_ (*r* = −0.303, *P *<* *0.0001) but not with Simoa amyloid-β markers. All measured individual LC-MS amyloid-β markers were moderately positively correlated (*r *=* *0.547–0.698, *P *<* *0.0001) and the LC-MS composite was strongly negatively correlated with LC-MS ln amyloid-β_1-42_/amyloid-β_1-40_ (*r* = −0.863, *P *<* *0.0001). Supplementary Table 3 provides Pearson correlations across all measured biomarkers with Bonferroni-corrected *P*-values.

### Associations of demographic factors with blood biomarkers

Significant sex differences were seen only for Simoa plasma amyloid-β_40_ [median (IQR), pg/ml: females 282 (255–315) versus males 296 (258–327), *P = *0.025] as shown in Supplementary Table 4.

All plasma amyloid-β species and ratios excepting Simoa plasma amyloid-β40 showed significant associations with age, even within the very narrow age range of this cohort. The direction of the association was inconsistent between the two methods; both Simoa plasma amyloid-β_42_ and Simoa plasma amyloid-β_42_/amyloid-β_40_ were positively associated with age, but the LC-MS measures were negatively associated with age (excepting the composite which had a positive association). For every year increase in age, there was a 0.1 log-fold decrease in LC-MS amyloid-β_1-42_/amyloid-β_1-40_, and a 0.2-fold increase in LC-MS composite. Simoa plasma p-tau181 was also positively associated with age, showing a 0.4 log-fold increase per year. These associations persisted despite adjustment for sex, *APOE* ε4 carrier status and SUVR, and were not attenuated by further adjustment for whole brain volume, subcortical white matter hyperintensity volume or PACC (Supplementary Table 5).

Considering cerebral amyloid as a continuous variable (SUVR), as expected higher SUVR was associated with lower Simoa plasma amyloid-β_42_ and amyloid-β_42_/amyloid-β_40_. Higher SUVR and being an *APOE* ε4 carrier were independently associated with lower LC-MS plasma amyloid-β_1-42_, amyloid-β_1-42_/amyloid-β_1-40_ and higher composite (Supplementary Table 6). A model incorporating age, sex, *APOE* ε4 carrier status and SUVR explained 1.2% of the variance in Simoa amyloid-β_42_, 3.4% for amyloid-β_42_/amyloid-β_40_ and 23.7% for p-tau181. For LC-MS methods this model explained 29.2%, 23.0% and 22.4% of the variance in amyloid-β_1-42_, amyloid-β_1-42_/amyloid-β_1-40_ and composite respectively.

There were no significant interactions (at *P *<* *0.05) between sex and SUVR, or sex and *APOE* ε4 carrier status, in their associations with the investigated blood biomarkers.

Time between blood sampling and scan did not have any significant associations with blood biomarkers, or confound their associations with SUVR.

### Concordance with amyloid PET status

Table 2 shows the AUC from ROC analyses for amyloid PET status in dementia-free individuals incorporating blood biomarkers as predictors, either alone or combined with age, sex and *APOE* ε4 carrier status. LC-MS amyloid-β_1-42_/amyloid-β_1-40_ (AUC 0.817, 95% confidence interval 0.770–0.864) and LC-MS composite (0.820, 0.775–0.866) performed significantly better than the best-performing Simoa biomarker (p-tau181: 0.707, 0.646–0.768; *P = *0.002 and *P *=* *0.001, respectively) and also significantly better than a model incorporating age, sex and *APOE* ε4 carrier status alone (0.695, 0.628–0.762; *P = *0.004 and *P *=* *0.002, respectively). Inclusion of these covariates did not significantly improve the performance of LC-MS amyloid-β_1-42_/amyloid-β_1-40_ or LC-MS composite. Sensitivity analyses in cognitively normal individuals without prior neurological conditions showed similar results (Supplementary Table 7). Combining Simoa p-tau181 with either Simoa or LC-MS amyloid-β did not contribute significantly to model performance (Supplementary Table 8). Further inclusion of educational attainment and time between blood sampling and scan also did not contribute significantly (data not shown). The best-performing Simoa and LC-MS biomarkers all had better concordance with amyloid PET status in *APOE* ε4 non-carriers than carriers (Supplementary Table 9).

**Table 2:** AUC from ROC analyses of amyloid PET status

Predictor(s)	Biomarker alone		**Biomarker + Age + sex + *APOE***ε**4 carrier status**	
	AUC	95% CI	AUC	95% CI
Age + sex + *APOE* ε4 carrier status	–	–	0.695	0.628, 0.762
Simoa plasma Aβ_42_/Aβ_40_	0.620	0.548, 0.691	0.727	0.665, 0.788
Simoa plasma p-tau181	0.707	0.646, 0.768	0.778	0.727, 0.828
LC-MS plasma Aβ_1-42_/Aβ_1-40_	0.817^a^^,b^	0.770, 0.864	0.841^c^	0.796, 0.886
LC-MS plasma composite	0.820^b^^,d^	0.775, 0.866	0.843^c^	0.798, 0.887

The results of analyses using the two best performing blood biomarkers from each platform are shown. Models incorporated blood biomarkers with and without inclusion of age, sex and *APOE* ε4 carrier status, in dementia-free individuals (*n *=* *441).

DeLong test results indicated where *P *<* *0.05:

^a^ *P* = 0.004 compared to age + sex + *APOE* ε4 carrier status; *P *=* *0.002 compared to Simoa plasma p-tau181.

^b^ *P* < 0.0001 compared to Simoa plasma Aβ_42_/Aβ_40_.

^c^ *P <* 0.0001 compared to age + sex + *APOE* ε4 carrier status.

^d^ *P* = 0.002 compared age + sex + *APOE* ε4 carrier status; *P* = 0.001 compared to Simoa plasma *p*-tau181.

Aβ = amyloid-β; AUC = area under the receiver operating characteristics curve; CI = confidence interval; p-tau181 = phospho-tau181.

**Figure 3:**
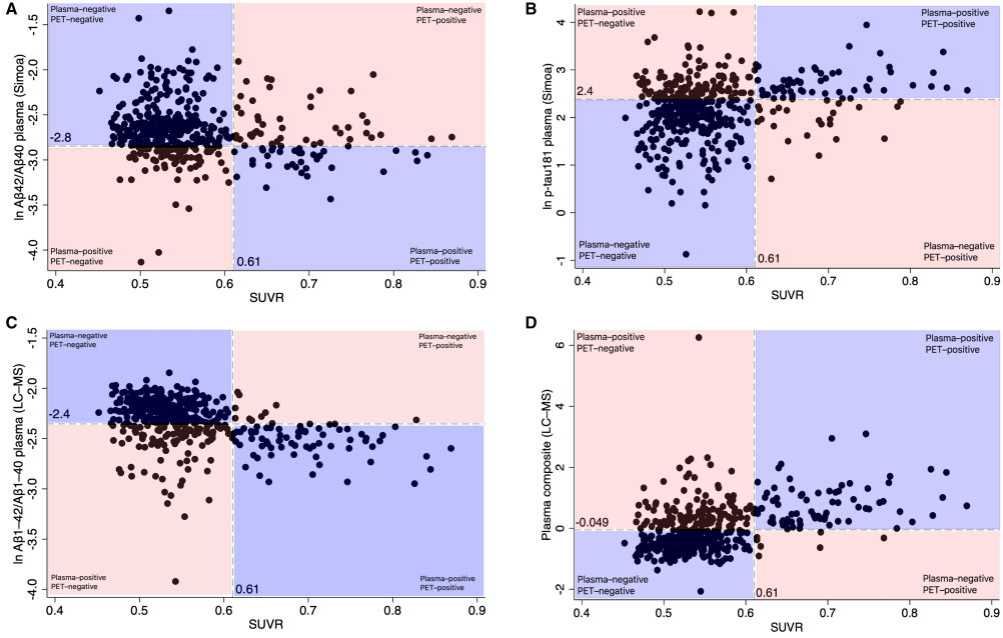
**Concordance of blood biomarkers with amyloid PET SUVR in dementia-free individuals in Insight 46 (*n *=* *441).** (**A**) ln Simoa plasma amyloid-β_42_/amyloid-β_40_. (**B**) ln Simoa plasma p-tau181. (**C**) ln LC-MS plasma amyloid-β_1-42_/amyloid-β_1-40_. (**D**) LC-MS plasma composite. Dashed vertical lines represent the SUVR cut-off point for PET positivity. Dashed horizontal lines represent the LC-MS biomarker cut-off points corresponding with Youden’s index derived by ROC analyses. Concordant classification by PET and plasma is represented by the blue area and discordant classification by the orange area on each graph. The non-log transformed cut-off points are 0.058 for Simoa plasma amyloid-β_42_/amyloid-β_40_, 10.8 pg/ml for Simoa plasma p-tau181, 0.095 for LC-MS plasma amyloid-β_1-42_/amyloid-β_1-40_ and −0.049 for LC-MS plasma composite. Aβ = amyloid-β; p-tau181 = phospho-tau181.

The percentage of individuals who were discordant for plasma and PET ranged from 26% to 32% depending on the plasma test used. Most discordant individuals were ‘plasma-positive, PET-negative’ using both the LC-MS biomarkers [104/115 = 90.4% of individuals discordant for LC-MS amyloid-β_42_/amyloid-β_40_ versus PET (Fig. 3C); and 124/130 = 94.6% of individuals discordant for the LC-MS composite versus PET (Fig. 3D)], but both types of discordance were seen using the Simoa biomarkers (Fig. 3A and B). The patterns were similar if different SUVR cut-off points were used (Supplementary Tables 10–13).

### Blood tests for screening prior to amyloid PET scan

Table 3 shows the potential outcomes of screening with the two best-performing biomarkers from each platform, in a population with amyloid PET positivity prevalence similar to Insight 46. Without plasma screening, 543 scans would be required to obtain 100 amyloid PET-positive individuals. Screening 940 individuals by using a base model of age, sex and *APOE* ε4 carrier status would reduce the number of scans required to 266 (i.e. by 50.0%). Neither Simoa plasma amyloid-β_42_/amyloid-β_40_ nor p-tau181 could outperform the base model, allowing for 41.3% and 44.4% reduction in scan numbers, respectively. However, screening 623 individuals with LC-MS amyloid-β_1-42_/amyloid-β_1-40_ using a cut-off point of 0.095 would reduce the number of scans required to 243 (i.e. by 54.4%) and screening 588 individuals with the LC-MS composite using a cut-off point of −0.049 would reduce the number of scans required to 264 (i.e. by 50.4%). Conversely, using multivariable models incorporating age, sex and *APOE* ε4 carrier status, LC-MS amyloid-β_1-42_/amyloid-β_1-40_ and composite would allow the number of scans to be reduced by 54.8% and 60.9%, respectively.

**Table 3:** Use of blood biomarkers for screening prior to amyloid PET scan

Model	Sensitivity, %	Specificity, %	Accuracy, %	Number needed to screen	Number proceeding to amyloid PET scan	Number of scans saved relative to no screening	% Scans saved relative to no screening	**% Scans saved relative to Age + sex + *APOE***ε**4 carrier status**
Age + sex + *APOE* ε4 carrier status	57.3	78.3	74.4	940	266	272	50.0	–
Simoa plasma Aβ_42_/Aβ_40_	45.1	78.0	71.9	1192	314	224	41.3	−17.6
Simoa plasma p-tau181	70.7	68.3	68.7	762	297	241	44.4	−11.4
LC-MS plasma Aβ_1-42_/Aβ_1-40_	86.6	71.9	74.6	623	243	295	54.4	8.5
LC-MS plasma composite	91.5	65.7	70.5	588	264	274	50.4	0.7
Simoa plasma Aβ_42_/Aβ_40_ + age + sex + *APOE* ε4 carrier status	68.3	72.1	71.4	794	280	258	47.4	−5.1
Simoa plasma p-tau181 + age + sex + *APOE* ε4 carrier status	90.2	52.4	59.4	596	331	207	38.1	−23.9
LC-MS plasma Aβ_1-42_/Aβ_1-40_ + age + sex + *APOE* ε4 carrier status	86.6	72.1	74.8	620	241	297	54.8	9.2
LC-MS plasma composite + age + sex + *APOE* ε4 carrier status	76.8	81.1	80.3	697	207	331	60.9	21.7

Values for sensitivity, specificity and accuracy were obtained by using Youden’s index cut-off points from each model for discriminating amyloid PET-positive from PET-negative dementia-free individuals (*n *=* *441). The penultimate column shows the percentage of scans saved relative to the number of scans that would be required without screening in a population with similar prevalence of amyloid PET-positivity to Insight 46 (18.6% prevalence of amyloid PET positivity would mean 538 scans would be required to obtain 100 amyloid PET-positive individuals). The last column shows the percentage of scans saved relative to the base model incorporating age + sex + *APOE* ε4 carrier status, where negative values indicate worse screening test performance than the base model. Aβ = amyloid-β; p-tau181 = phospho-tau181.

**Figure 4:**
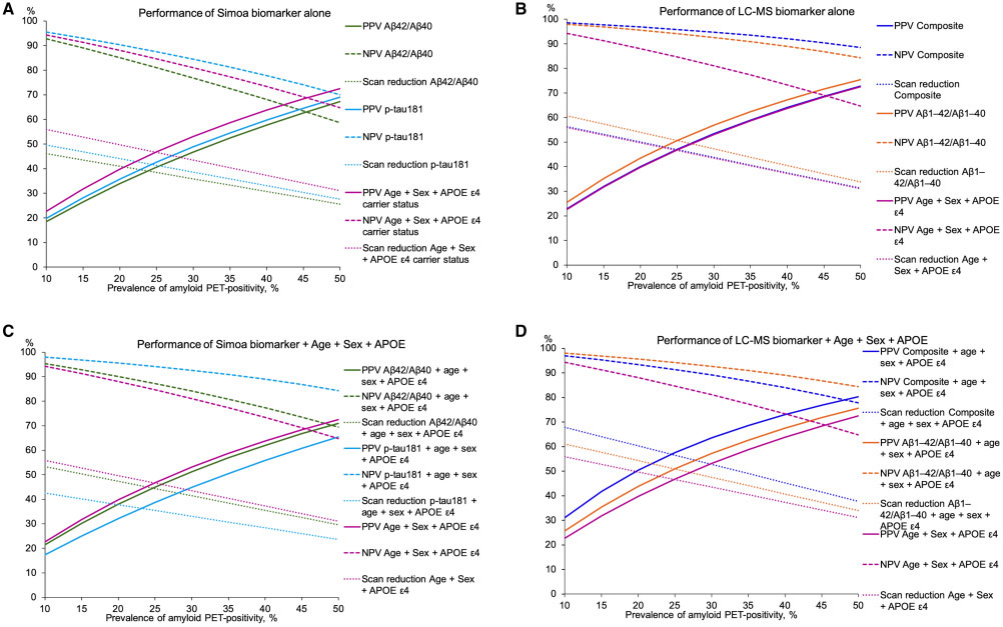
**Hypothetical performance of the best-performing Simoa and LC-MS tests by prevalence of amyloid PET positivity.** (**A**) Simoa biomarker alone. (**B**) LC-MS biomarker alone. (**C**) Simoa biomarker + age + sex + *APOE* ε4 carrier status. (**D**) LC-MS biomarker + age + sex + *APOE* ε4 carrier status. Lines were modelled by computing the positive (PPV) and negative predictive values (NPV) of the tests and the scan reduction afforded at specified values of amyloid PET positivity prevalence over the range 10–50%, in 5% intervals. Scan reduction is the percentage of scans saved relative to the number of scans that would be required without screening (calculated according to the specified prevalence of amyloid PET positivity). Solid lines show PPV, dashed lines show NPV and dotted lines show scan reduction. Aβ = amyloid-β; p-tau181 = phospho-tau181.

Extending the findings to hypothetical populations with differing prevalence of amyloid PET positivity, Fig. 4 shows that regardless of the screening test used, greater scan reduction would be afforded in populations with lower prevalence of amyloid PET positivity. Of the two Simoa platform tests, only plasma p-tau181 would have better negative predictive value (NPV) than the base model, but neither Simoa test would perform better than the base model in terms of positive predictive value (PPV) or scan reduction (Fig. 4A). The greatest PPV and scan reduction would be afforded by LC-MS amyloid-β_1-42_/amyloid-β_1-40_, while the NPV would be greatest for the LC-MS composite (Fig. 4B). If added to age, sex and *APOE* ε4 carrier status, LC-MS composite would have an improvement in PPV and scan reduction at the expense of a worsening of NPV, whereas LC-MS amyloid-β_1-42_/amyloid-β_1-40_ would have an improvement in NPV without much change in PPV or scan reduction (Fig. 4D).

### Relative costs of blood screening

**Figure 5:**
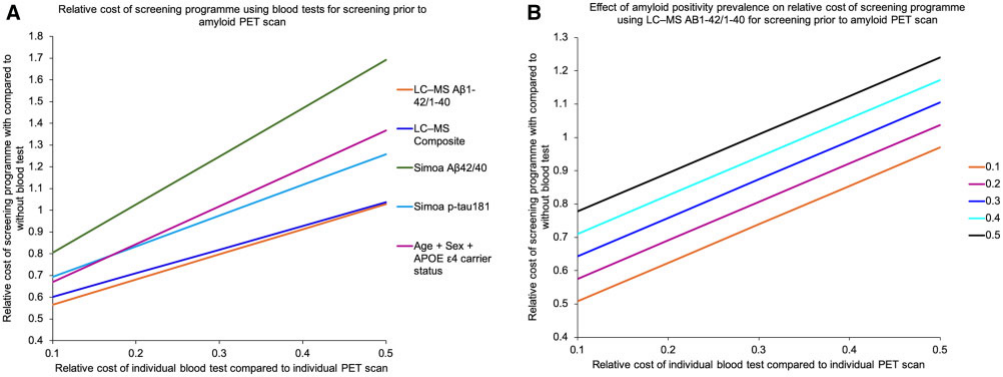
**Relative costs of screening programmes using different blood tests.** (**A**) Using different blood tests, at fixed prevalence of amyloid PET positivity. (**B**) Using LC-MS plasma amyloid-β_1-42_/amyloid-β_1-40_ over a range of 10–50% of theoretical prevalence of amyloid PET positivity. Aβ = amyloid-β; p-tau181 = phospho-tau181.

Using the equations in Fig. 1, in a population of similar prevalence of amyloid PET positivity to Insight 46, the LC-MS amyloid-β_1-42_/amyloid-β_1-40_ test would yield the lowest relative cost of screening with versus without using blood tests (Fig. 5A) across a range of theoretical fractional costs of individual blood tests compared to PET scans. However, with higher prevalence of amyloid PET positivity, as may be seen with increasing age, the screening programme would be rendered less economical for a given blood test (Fig. 5B shows the relationship with amyloid PET positivity for the LC-MS amyloid-β_1-42_/amyloid-β_1-40_ test).

## Discussion

In more than 400 dementia-free individuals of near-identical age, we show that LC-MS methods for measuring plasma amyloid-β_1-42_/amyloid-β_1-40_ and a plasma composite outperform the Simoa amyloid-β_42_/amyloid-β_40_ and p-tau181 assays in their ability to discern amyloid PET status. Using either LC-MS method to screen before PET scanning has the potential to yield significant savings for clinical trial recruitment, affording further reductions in the required number of PET scans compared to the number of scans needed without pre-screening or when using age, sex and *APOE* ε4 carrier status for screening.

Despite our cohort’s narrow age range, lower LC-MS amyloid-β_1-42_/amyloid-β_1-40_ was associated with increased age, and this was not attenuated by further adjustment for PACC, white matter hyperintensity volume or whole brain volume. A more age-diverse study (Schindler *et al.*, 2019) has reported a negative association, but its magnitude was much smaller (a reduction of ∼3% per year of age). In our age-restricted study it is therefore possible that this finding either represents a true age effect, or reflects some other source of variation between those attending near the start and the end of the study (James *et al.*, 2018), or both.

We found associations between *APOE* ε4 carrier status and LC-MS amyloid-β_1-42_ and amyloid-β_1-42_/amyloid-β_1-40_ independent of SUVR. However, while other groups using mass spectrometry-based techniques for quantifying plasma amyloid-β to predict cerebral amyloid status (Ovod *et al.*, 2017; Nakamura *et al.*, 2018; Schindler *et al.*, 2019) commonly included age and *APOE* ε4 carrier status in predictive models, we found that including these variables made no material difference to the ability to predict amyloid status when using LC-MS amyloid-β_1-42_/amyloid-β_1-40_, and only small improvements using the LC-MS composite proposed by Nakamura *et al.* (2018). Noting that amyloid PET results are not routinely adjusted for *APOE* ε4 carrier status, this provides further confidence that the LC-MS assay is directly reflecting brain amyloid-β. Our finding that the majority of discordant cases were ‘plasma-positive, PET-negative’, and this persisted despite changing the PET-positivity cut-off, is consistent with prior studies showing similar results for plasma (Schindler *et al.*, 2019) and CSF (Palmqvist *et al.*, 2016), suggesting that as with CSF, plasma amyloid-β markers may become abnormal before a threshold for cortical amyloid-β positivity is reached.

Our results were not significantly altered by using different PET cut-off points. However, Youden’s index cut-off points for plasma amyloid-β, which we chose to maximize the combination of sensitivity and specificity, may not be the most useful in every screening setting. For example, in recruitment to a clinical trial it may be desirable to choose a cut-off point that maximizes sensitivity, although this increases the false positive rate. In contrast, for population screening for clinical purposes it may be desirable to minimize false positives, even if at the expense of the false negative rate.

In addition to cost reduction, plasma testing may allow for screening of more diverse populations at scale, and reductions in screen failures, resulting in faster clinical trial recruitment. We demonstrate that the greatest potential savings would be made at lower prevalence of amyloid-β positivity. The prevalence of amyloid PET positivity is highly age-dependent; as shown in a 2015 meta-analysis it was 16% at 60 years, 23% at 70 years and 33% at 80 years (Jansen *et al.*, 2015). In more recent data from the Mayo Clinic Study of Aging the prevalence was 18% at age 60–69 and 32% at age 70–79 (Roberts *et al.*, 2018) and the former figure is consistent with the prevalence of 18.6% we note in Insight 46. As with any screening test, false positives will be expected, and a general consideration with all amyloid biomarker studies of asymptomatic individuals is that progression from amyloid positivity to clinical signs or symptoms may take many years (Vos *et al.*, 2013; Donohue *et al.*, 2017; Roberts *et al.*, 2018), so amyloid-positive individuals may never develop cognitive symptoms in their lifetime. Any use of plasma biomarker-based screening will require clear protocols for counselling and communicating plasma test results to prospective participants (Harkins *et al.*, 2015), including that a positive result is likely to require confirmation with another more definitive modality (PET or CSF).

The accuracy of the Simoa plasma p-tau181 assay in predicting amyloid PET status in Insight 46 was lower than that previously published in cohorts comprising mixed populations of cognitively unimpaired individuals, MCI and Alzheimer’s disease dementia; the AUC were 0.707 in Insight 46, 0.881 in the Translational Biomarkers of Aging and Dementia (TRIAD) cohort and 0.761 in the BIOFINDER-2 cohort in which the same assay was used (Karikari *et al.*, 2020), and 0.79–0.81 in the BioFINDER-2 cohort in which a different Mesoscale Discovery assay was used (Janelidze *et al.*, 2020). In the TRIAD cohort, when analysis was limited to cognitively unimpaired older individuals, the AUC for prediction of amyloid PET status was 0.772. The differences observed may relate to differences in amyloid PET tracers (^18^F-florbetapir in Insight 46, ^18^F-AZD-4694 in TRIAD and ^18^F-flutemetamol in BioFINDER) but may also be influenced by differences in the cohorts in terms of burden of tau pathology, which is also highly correlated with plasma p-tau181 (Mielke *et al.*, 2018; Janelidze *et al.*, 2020; Karikari *et al.*, 2020). As these cohorts had wider age ranges, including some individuals up to a decade older than those in Insight 46, amyloid PET-positive individuals in those cohorts may have had a greater burden of cerebral tau pathology at the time of blood sampling than those in Insight 46, resulting in higher plasma p-tau181 levels. At this phase of Insight 46 we did not have a measure of cerebral tau pathology such as tau PET, and therefore were not able to investigate the relative predictive capacity of plasma p-tau181 for amyloid PET versus tau PET. Since performing our study, an immunoassay for plasma p-tau217 has been developed that shows improved predictive capacity for neuropathologically defined Alzheimer’s disease versus non-Alzheimer’s disease dementias (AUC 0.98 for plasma p-tau217 and 0.85 for plasma p-tau181), improved performance for biomarker-supported *in vivo* diagnosis of Alzheimer’s disease versus non-Alzheimer’s disease (AUC 0.96 and 0.81 respectively), good concordance with cerebral tau deposition defined by tau PET, and elevations in presenilin-1 mutation carriers with presymptomatic dominantly inherited Alzheimer’s disease compared to non-carriers (Palmqvist *et al.*, 2020). A mass spectrometry-based plasma p-tau217 assay (Barthélemy *et al.*, 2020) has also shown good predictive capacity for CSF amyloid status in two smaller cohorts consisting of a mixture of younger and older controls, and individuals with cognitive impairment with and without CSF amyloid pathology (AUC 0.92–0.99 for plasma p-tau217 as opposed to 0.75–0.98 for plasma p-tau181). Future work will therefore benefit from comparing the plasma p-tau217 and p-tau181 assays in their utility for predicting amyloid status in preclinical cohorts like Insight 46.

While our study design has strengths, including general population-representativeness at initial recruitment, direct comparison of blood testing platforms, and concurrent prospectively-collected plasma and PET amyloid data, we acknowledge certain limitations. Our participants are exclusively white British, so these findings cannot be extrapolated directly to ethnically diverse populations. The narrow age range of our study might also have underestimated predictive capacity of models of amyloid PET status that incorporated age, sex and *APOE* ε4 carrier status. Using global cortical amyloid PET as an outcome may underestimate the true number of participants with cerebral amyloid deposition, as CSF changes and region-specific amyloid PET deposition (Palmqvist *et al.*, 2016), may occur earlier than when participants become ‘amyloid PET-positive’ by global cortical PET SUVR. CSF was not available for this phase of data collection in Insight 46. Using CSF as the comparator instead of PET might reduce the cost savings of blood tests, as CSF testing is likely to be more economical than PET, both in terms of initial setup and ongoing costs (Wittenberg *et al.*, 2019). Our assessment of the relative costs of screening using blood tests is simplified, as it absorbs any differences in setup costs between the two blood test platforms into one theoretical cost estimate for each test. Both platforms require expensive instruments and specialist operators, and it is likely that real-world differences in setup and maintenance costs would play an important role in choosing one platform over another, in addition to differences in accuracy between platforms.

In conclusion, we demonstrate superior performance of the LC-MS platform over Simoa assays for plasma amyloid-β and p-tau181, and over a model incorporating age, sex and *APOE* ε4 carrier status, in screening for concurrent PET amyloid status in a large number of dementia-free individuals. Our study strengthens a growing body of evidence that plasma screening can reduce the numbers of amyloid PET scans required to identify amyloid-β-positive individuals, for recruitment to clinical trials or ultimately for giving anti-amyloid therapies, and suggests that this may be feasible in a preclinical cohort.

## Supplementary Material

awaa403_Supplementary_Data

## Abbreviations

LC-MS: liquid chromatography-mass spectrometry
MCI: mild cognitive impairment
Simoa: single molecule array
SUVR: standardized uptake value ratio
