# Supplementary material for: Population-based blood screening for preclinical Alzheimer’s disease in a British birth cohort at age 70
Source: Brain. 2021 Jan 22;144(2):434–49. doi: 10.1093/brain/awaa403 (PMC7940173; doi:10.1093/brain/awaa403)
Supplement: awaa403_Supplementary_Data [file awaa403_supplementary_data.pdf]

Supplementary Appendix

for

Population-based pre-screening for asymptomatic cerebral amyloid-β pathology: a comparison of two plasma-based methods in a British birth cohort (Keshavan *et al.*)

Supplementary methods

Simoa measurements

Plasma Aβ40 and Aβ42

A single 0.5 mL aliquot of plasma for each individual was thawed directly to room temperature over 1 hour and vortexed for 2 seconds. 0.3 mL was pipetted into a 1.5 mL polypropylene centrifuge tube and centrifuged at 13 000 g for 10 minutes as per the kit manufacturer's recommendation; the remaining 0.2mL was replaced into -80 ºC in the original cryovial. After the 0.3 mL was centrifuged, 0.1mL of the supernatant was pipetted onto each of two plates for analysis in duplicate, capitalising on the ability to load two different reagent kits at a time on the HD-1 analyser. When plates were prepared in this way, the plate containing samples for Aβ40 was always analysed first, and that containing samples for Aβ42 was analysed second. The CV across the duplicates was <15% for all samples assayed for Aβ40, but for some samples assayed for Aβ42 the CV was >15% or no value was returned. In this case the procedure above was repeated at a later date, using a fresh 0.5 mL aliquot of plasma and pipetting out and centrifuging 0.2 mL, then pipetting 0.1 mL of the supernatant onto the plate for Aβ42 analysis.

After thawing directly to room temperature over an hour, vortexing for 2 seconds and then pipetting 0.2 mL into a 1.5 mL polypropylene centrifuge tube, samples were centrifuged at 13000 g for 10 minutes, as per the kit manufacturer's recommendation. 0.13 mL of the supernatant was pipetted onto the plate for analysis in duplicate, using commercially available Simoa tau kits of the same batch. However, if the Aβ42 assay was being repeated on the second aliquot, then on the same thaw of this sample, after vortexing for 2 seconds, 0.2 mL was pipetted into a separate 1.5 mL polypropylene centrifuge tube and used for plasma t-tau analysis in parallel. In this situation, the plate of samples for analysis of Aβ42 was analysed first and the plate for analysis of t-tau second. If the CV across duplicates was >15% on the first analysis of t-tau, the analysis was repeated at a later date, employing one additional freeze-thaw cycle by starting with the 0.3 mL volume that was in the original cryovial.

By this method, all samples analysed for plasma Aβ40 and Aβ42 underwent one freeze-thaw cycle. Of 498 available plasma samples, quantification with a CV <15% across duplicates was achieved for 497 for Aβ42 and 496 for Aβ40. Inter-assay CV for 2 run validation controls were 6% and 8% for Aβ40, and 22% and 28% for Aβ42.

Plasma p-tau181

A 0.5 mL aliquot of plasma for each individual was transported on solid carbon dioxide to the University of Gothenburg, Sweden; samples were immediately stored at -80 ºC until use. On the day of the analysis, the blood samples were allowed to thaw at room temperature for an hour, vortexed at 200 rpm for 30 s, and centrifuged at 4000 g for 10 min, then 90 µL of the supernatant was used for p-tau181 measurements. Separate anonymized plasma samples from the University of Gothenburg, pooled to give two different p-tau181 concentrations, were used as internal quality control (iQC) samples, and were processed identically to the test samples. Each iQC sample was analyzed in duplicate at the start and the end of each plate. Combined iQC data from eight separate runs were used to determine the within- and between-run variations, following the recommendations of an international group of neurochemists (Andreasson *et al.*, 2015).

The high-concentration iQC sample (concentration expressed in mean ± standard deviation = 17.1 ± 0.7 pg/mL) had a within-run variation of 0.4% and a between-run variation of 10.3%. For the low-concentration iQC sample (concentration = 4.8 ± 0.5 pg/mL), the within- and between-run variations were 10.9% and 13.0% respectively. Identical batches of assay reagents were used throughout the measurements for this cohort.

Extended immunoprecipitation liquid chromatography–mass spectrometry methods

Sample preparation

One 0.5 mL aliquot of plasma from each individual was shipped on solid carbon dioxide to the University of Gothenburg.

Fifty µL Dynabeads™ M-280 Sheep Anti-Mouse IgG magnetic beads (6–7×10^8 beads/mL, ~10 mg/mL) per sample were transferred to a polypropylene (tube. The tube was placed in a magnet stand for one minute and the supernatant was then discarded. The beads were then washed three times in twice the original volume with phosphate-buffered saline (PBS, pH 7.4) before re-suspending the beads in the original volume of PBS. The washed beads were divided to two separate polypropylene tubes.

Purified anti-β-Amyloid 17-24 (4G8) and anti-β-Amyloid 1-16 antibodies (6E10, both Biolegend, San Diego, CA) were added to the magnetic beads at a final concentration of 137 µg/mL, each in a separate tube and mixed for one hour on a roller at +20 °C. The beads were washed three times in twice the original volume and then suspended in the original volume with PBS prior to combining the content of the two tubes.

Recombinant ^{15}N uniformly labelled B-Amyloid (Aβ) peptides 1-38, 1-40 and 1-42 (rPeptide, Watkinsville, GA) were used as internal standards (IS) and prepared at 2.7 ng/mL in 76/20/4 (volume/volume) ultrapure water: acetonitrile: concentrated ammonia (~25 %). Calibrators were prepared using recombinant Aβ1-38, Aβ1-40 and Aβ1-42 (rPeptide, Watkinsville, GA) in an artificial matrix consisting of 8 % bovine serum albumin (BSA) in PBS (weight/weight%) at six equidistant levels from 5 to 100 pg/mL for Aβ1-38 and Aβ1-42 and 20 to 400 pg/mL for Aβ1-40.

Plasma samples were centrifuged at 2500 RCF for 10 minutes at +4 °C directly after thawing at room temperature. 250 µL of each sample and calibrator was transferred to a KingFisher deep-well 96 plate (Thermo Scientific, #95040450). 20 µL of the 2.7 ng/mL IS solution was added to all samples (including calibrators) giving a final concentration of 200 pg/mL in

samples. The samples were diluted with 660 µL PBS and the sample plate was placed in a KingFisher™ Flex Purification System (Thermo Fisher Scientific, #5400630), where the samples were mixed for 20 minutes. After removing the plate, 20 µL of 10 % Triton X-100 and 50 µL of magnetic beads with bound antibodies were added to each well and the plate was placed in the KingFisher™ Flex. After the samples and beads were mixed for 1.5 hours, the beads from each well were washed in 1 mL 0.2 % Triton x100 in PBS followed by 1 mL PBS and finally 1 mL 50 mM ammonium bicarbonate for 10 seconds in each solution. Finally. the Aβ peptides were eluted from the antibodies by mixing the beads from each sample in 0.1 mL 0.5 % formic acid in water (in a KingFisher 96 KF microplate 200µL, Thermo Fisher Scientific, #97002540) for four minutes. The eluates were dried using vacuum centrifugation using a Savant SC210A SpeedVac Concentrator (Thermo Fisher Scientific, #SC210A-230) without applying heat.

LC-MS/MS

Mobile phases for the liquid chromatography (LC) were prepared using ultrapure (type 1) water from a Merck Synergy UV water purification system (Merck, #SYNSVHFWW) set to a water resistivity of 18.2 MΩ.cm at 25 °C, Acetonitrile (ACN), Far UV HPLC Gradient grade (Fisher Scientific, #A/0627/17X) and Ammonium hydroxide solution, puriss. p.a., reag. ISO, reag. Ph. Eur., ~25% NH_3 basis (Sigma-Aldrich, #30501-1L-D). Mobile phase A consisted of 5 % ACN and 0.3 % concentrated ammonia solution (v/v) in water and mobile phase B consisted of 4 % water and 0.1 % (v/v) concentrated ammonia solution in ACN. Wash solution consisted of 50 % ACN and 4 % concentrated ammonia solution (volume/volume) in water. LC was performed using an UltiMate™ 3000 system (Thermo Fisher Scientific). The analytical columns were a Proswift RP-4H 1×250 mm monolithic column (Thermo Fisher Scientific, #066640) maintained at 50 °C. A dual pump and column approach was used to increase throughput of the method, where one pump eluted the analyte from one column as the other pump equilibrated the second column before switching columns using a 10-port valve.

The dried samples were dissolved in 50 µL 20 % ACN and 4 % concentrated ammonia solution (volume/volume) in water and placed on shaker for 15 minutes at 600 rpm. The samples were then placed in an autosampler. After injection of 40 µL of the dissolved sample, gradient elution was performed with one pump at a flow rate of 0.3 mL/min with the following gradient steps: 0 min, 5 % B; 3 min, 20 % B; 3.5 min, 95 % B; 3.9 min, 95 % B; 4 min, 5 % B. The second pump simultaneously equilibrated the second column at a flow rate of 0.3 mL/min with the following gradient steps: 0 min, 95 % B; 2 min, 95 % B; 3 min, 5 % B; 5 min, 5 % B. The auto sampler injector needle and tubing were washed with wash solution after each sample injection.

Mass spectrometric analysis was performed using a quadrupole-Orbitrap hybrid mass spectrometer (Q-Exactive) equipped with a heated electrospray ionization source (HESI-II) (Thermo Fisher Scientific, Bremen, Germany) using the following ion source parameters: probe position D, sheath gas 35, auxiliary gas 15, spray voltage 4.40 kV, S-lens RF 55, heater +350 °C, and capillary temperature +320 °C. The precursors were isolated with isolation windows of 2.5 m/z followed by fragmentation using a normalized collision energy of 19.0. Parallel reaction monitoring (PRM) was performed by recording fragment spectra at a resolution of 17.500 using an automatic gain control (AGC) target of 5×10^5 charges and a maximum injection time of 250 ms. Ion chromatograms were constructed by summing the peak areas of selected ions fragment of the 4+ charge states for the respective Aβ peptides and their corresponding internal standards (Supplementary table 1). The PRM method was

scheduled to scan for one Aβ peptide and its internal standard at a time to maximize sensitivity.
Data processing and quantification was performed using Thermo Xcalibur 4.1.

Supplementary results

List of supplementary tables

Supplementary table 1: Precursor and product ions for Aβ peptides used for the parallel reaction monitoring method. 6

Supplementary table 2: Comparison of characteristics between dementia-free individuals with missing amyloid PET scan and blood biomarker data and those who had useable amyloid scans and complete blood biomarker data. 8

Supplementary table 3: Inter-biomarker correlations in dementia free-individuals with full blood biomarker and amyloid PET data (n = 441). 9

Supplementary table 4: Unadjusted differences in blood biomarkers by sex in dementia-free individuals, n=441. 10

Supplementary table 5: Associations of blood biomarkers with age in dementia-free individuals (n=441). 11

Supplementary table 6: Associations of blood biomarkers with SUVR and APOE ε4 carrier status, adjusted for age and sex, in dementia-free individuals (n=441). 13

Supplementary table 7: Areas under the curve from ROC analyses of amyloid PET status incorporating blood biomarkers, with and without inclusion of age, sex and APOE ε4 carrier status, in cognitively normal individuals without prior neurological conditions (n=410). 14

Supplementary table 8: Areas under the curve from ROC analyses of amyloid PET status incorporating combinations of plasma biomarkers, with and without inclusion of age, sex and APOE ε4 carrier status, in dementia-free individuals (n=441). 15

Supplementary table 9: Subgroup analysis: areas under the curve from ROC analyses of amyloid PET status incorporating individual biomarkers, in dementia-free APOE ε4 non-carriers (n = 315) and carriers (n = 126). 16

Supplementary table 10: Influence of SUVR cut-point for amyloid PET status on Simoa Aβ42/Aβ40 performance in dementia-free individuals in Insight 46 (n=441). 17

Supplementary table 11: Influence of SUVR cut-point for amyloid PET status on Simoa p-tau181 performance in dementia-free individuals in Insight 46 (n=441). 18

Supplementary table 12: Influence of SUVR cut-point for amyloid PET status on LC–MS Aβ1–42/1–40 performance in dementia-free individuals in Insight 46 (n=441). 19

Supplementary table 13: Influence of SUVR cut-point for amyloid PET status on LC–MS composite performance in dementia-free individuals in Insight 46 (n=445). 20

Supplementary tables

Precursor ion	Product ions m/z (ion type and charge state)
native Aβ1-42 (m/z 1129.58, charge state: 4+)	915.19 ($b33^{4+}$), 943.21 ($b34^{4+}$), 975.98 ($b35^{4+}$), 1000.74 ($b36^{4+}$), 1029.51 ($b38^{4+}$), 1054.03 ($b39^{4+}$), 1078.79 ($b40^{4+}$), 1107.06 ($b41^{4+}$), 1163.23 ($b31^{3+}$), 1200.25 ($b32^{3+}$), 1257.29 ($b34^{3+}$), 1300.96 ($b35^{3+}$), 1333.66 ($b36^{3+}$), 1372.00 ($b38^{3+}$), 1405.02 ($b39^{3+}$)
15N-Aβ1-42 (m/z 1143.00, charge state: 4+)	915.19, 943.21, 975.98, 1000.74, 1029.51, 1054.03, 1078.79, 1107.06, 1163.23, 1200.25, 1257.29, 1300.96, 1333.66, 1372.00, 1405.02
native Aβ1-40 (m/z 1083.47, charge state: 4+)	915.00($b33^{4+}$), 943.29($b34^{4+}$), 976.09($b35^{4+}$), 1000.87($b36^{4+}$), 1015.13($b37^{4+}$), 1029.40($b38^{4+}$), 1054.18($b39^{4+}$), 953.69($b24^{3+}$), 1125.20($b30^{3+}$), 1162.92($b30^{3+}$), 1200.64($b32^{3+}$), 1219.66($b33^{3+}$), 1257.38($b34^{3+}$), 1301.11($b35^{3+}$), 1334.16($b36^{3+}$), 1353.17($b37^{3+}$), 1372.19($b38^{3+}$)
15N-Aβ1-40 (m/z 1096.63, charge state: 4+)	926.42, 954.96, 988.96, 1013.04, 1027.55, 1042.06, 1067.09, 965.28, 1139.44, 1177.49, 1215.54, 1234.89, 1272.94, 1317.01, 1350.38, 1369.73, 1389.08
native Aβ1-38 (m/z 1033.90, charge state: 4+)	915.00($b33^{4+}$), 943.29($b34^{4+}$), 976.09($b35^{4+}$), 1000.87($b36^{4+}$), 1015.13($b37^{4+}$), 1125.21($b30^{3+}$), 1162.92($b31^{3+}$), 1200.64($b32^{3+}$), 1219.66($b33^{3+}$), 1257.38($b34^{3+}$), 1301.11($b35^{3+}$)
15N-Aβ1-38 (m/z 1046.57, charge state: 4+)	926.42, 954.96, 988.01, 1013.04, 1027.55, 1139.44, 1177.50, 1215.55, 1234.89, 1272.94, 1317.01
native Aβ(-3)-40 (m/z 1173.09, charge state: 4+)	1004.62($b36^{4+}$), 1032.91($b37^{4+}$), 1065.71($b38^{4+}$), 1090.50($b39^{4+}$), 1104.76($b40^{4+}$), 1119.02($b41^{4+}$), 1143.80($b42^{4+}$), 1244.71($b33^{3+}$), 1282.43($b34^{3+}$), 1320.15($b35^{3+}$), 1339.16($b36^{3+}$), 1376.88($b37^{3+}$), 1420.61($b38^{3+}$), 1453.66($b39^{3+}$), 1491.69($b41^{3+}$)

Supplementary table 1: Precursor and product ions for Aβ peptides used for the parallel reaction monitoring method.

The ion types and charge states are identical for each native peptide and its corresponding internal standard (^{15}N-labelled).

Abbreviations: Aβ, amyloid-β; m/z, mass to charge ratio.

	All dementia-free with amyloid scan and full blood biomarker data (n = 441)	Dementia-free with amyloid scan but missing blood biomarker data (n = 18)*	Dementia-free with full blood biomarker data but missing amyloid scan (n = 35)*	Dementia and/or missing amyloid scan and/or missing blood biomarker data (n = 61)*
**Characteristic**	Value	Value	Value	Value
Age at blood sampling, y	70.7 (0.7)	70.4 (0.7)	70.7 (0.7)	70.6 (0.7)
Sex, % female	50.6	38.9	48.6	44.3
APOE ε4 status, % carrier	28.6	43.8	34.3	37.3
MMSE	30 (29, 30)	29 (29, 30)	30 (29, 30)	30 (29, 30)
PACC (z score)	0.015 (0.682)	0.029 (0.737)	0.079 (0.711)	-0.126 (1.032)
Educational attainment by age 26, n (%)				
No qualification	65 (14.7)	5 (27.8)	4 (11.4)	13 (21.3)
Vocational	23 (5.2)	0	3 (8.6)	3 (4.9)
O-Level/equivalent	112 (25.4)	5 (27.8)	8 (22.9)	13 (21.3)
A-Level/equivalent	159 (35.8)	5 (27.8)	13 (37.1)	21 (34.4)
Degree/equivalent	83 (18.8)	3 (16.7)	7 (20.0)	11 (18.0)
Number (%) individuals with blood sample and amyloid scan not done on same day	59 (13.4)	3 (16.7)	1 (2.9)	4 (6.6)
Delay between blood sample and amyloid scan for individuals who did not have them on the same day, y	0.131 (0.060, 0.211) n=59	0.170 (0.057, 0.591)	0.079^{+}	0.125 (0.063, 0.486)
Total intracranial volume, mL	1427 (1341, 1517)	1405 (1329, 1496)	1505 (1458, 1638)	1448 (1344, 1570) n = 29
Whole brain volume, mL	1100 (1034, 1162) n = 439	1074 (1025, 1160)	1165 (1114, 1235) n = 7	1113 (1039, 1193) n = 29
White matter hyperintensity volume, mL	3.1 (1.6, 6.8) n = 427	2.7 (1.9, 6.8)	4.3 (2.5, 8.8) n = 7	3.7 (2.3, 7.6) n = 30
Serum creatinine, µmol/L	73 (64, 84)	78 (62, 87) n = 14	81 (70, 92)	82 (69, 92) n = 56
Body mass index	27.3 (24.3, 30.2)	26.7 (23.0, 31.7)	28.6 (25.7, 31.5)	28.9 (25.2, 31.8)
Simoa plasma Aβ40, pg/mL	288 (256, 322)	278 (228, 341) n = 13	281 (261, 335)	284 (260, 335) n = 55
Simoa plasma Aβ42, pg/mL	19.6 (16.7, 22.7)	18.6 (15.7, 27.4) n = 14	19.9 (16.1, 23.2)	18.9 (15.7, 22.9) n = 56
Simoa plasma Aβ42/Aβ40	0.066 (0.058, 0.077)	0.064 (0.056, 0.117) n = 13	0.068 (0.060, 0.076)	0.066 (0.057, 0.076) n = 55
Simoa plasma p-tau181, pg/mL	9.2 (6.4, 12.9)	6.1 (4.3, 8.4) n = 11	8.3 (5.5, 11.7)	8.1 (5.5, 12.1) n = 50
LC–MS plasma Aβ1–38, pg/mL	24.8 (21.6, 28.0)	23.3 (22.3, 26.9) n = 11	25.2 (21.1, 29.2)	25.2 (21.8, 28.5) n = 52
LC–MS plasma Aβ1–40, pg/mL	284 (257, 314)	280 (264, 304) n = 9	302 (237, 332)	287 (253, 331) n = 50

LC–MS plasma Aβ1–42, pg/mL	28.6 (23.4, 33.3)	29.1 (26.2, 35.1) n = 11	27.8 (22.8, 39.4)	28.2 (24.0, 35.6) n = 52
LC–MS plasma Aβ3–40, pg/mL	30.1 (24.4, 35.7)	28.5 (26.2, 32.6) n = 11	33.2 (26.5, 38.1)	31.1 (26.3, 38.0) n = 52
LC–MS plasma Aβ1–42/Aβ1–40	0.099 (0.087, 0.113)	0.103 (0.094, 0.119) n = 8	0.100 (0.089, 0.115)	0.100 (0.090, 0.114) n = 49
LC–MS plasma composite	-0.001 (0.799)	0.011 (1.049) n = 8	0.005 (1.057)	0.035 (1.021) n = 49

Supplementary table 2: Comparison of characteristics between dementia-free individuals with missing amyloid PET scan and blood biomarker data and those who had useable amyloid scans and complete blood biomarker data.

Values are expressed as mean (standard deviation) for normally distributed variables, median (interquartile range) for skewed variables, and percentages for binary variables.
* number unless otherwise stated
$^{+}$ single exact value given

Abbreviations: Aβ, amyloid-β; *APOE* ε4, apolipoprotein E gene epsilon 4 allele; LC–MS, liquid chromatography–mass spectrometry; MMSE, mini-mental state examination; NSHD, National Survey of Health and Development; PACC, pre-clinical Alzheimer's cognitive composite; p-tau181, phospho-tau181; SD, standard deviation; SUVR, standardised uptake value ratio.

r<br>*P* (Bonferroni)	ln Aβ40<br>(Simoa)	ln Aβ42<br>(Simoa)	ln Aβ42/<br>Aβ40 (Simoa)	ln p-tau181<br>(Simoa)	ln Aβ1–38<br>(LC–MS)	ln Aβ1–40<br>(LC–MS)	ln Aβ1–42<br>(LC–MS)	ln Aβ-3–40<br>(LC–MS)	ln Aβ1–42/<br>Aβ1–40<br>(LC–MS)
ln Aβ42<br>(Simoa)	**0.254**<br>**<0.0001**								
ln Aβ42/ Aβ40<br>(Simoa)	**-0.307**<br>**<0.0001**	**0.843**<br>**<0.0001**							
ln p-tau181<br>(Simoa)	0.109<br>0.993	-0.003<br>1.000	-0.063<br>1.000						
ln Aβ1–38<br>(LC–MS)	**0.354**<br>**<0.0001**	0.066<br>1.000	-0.132<br>0.249	-0.082<br>1.000					
ln Aβ1–40<br>(LC–MS)	**0.406**<br>**<0.0001**	0.110<br>0.950	-0.118<br>0.594	0.056<br>1.000	**0.698**<br>**<0.0001**				
ln Aβ1–42<br>(LC–MS)	**0.232**<br>**<0.0001**	**0.207**<br>**0.001**	0.074<br>1.000	**-0.204**<br>**0.001**	**0.636**<br>**<0.0001**	**0.610**<br>**<0.0001**			
ln Aβ-3–40<br>(LC–MS)	**0.253**<br>**<0.0001**	0.121<br>0.493	-0.022<br>1.000	-0.010<br>1.000	**0.547**<br>**<0.0001**	**0.570**<br>**<0.0001**	**0.691**<br>**<0.0001**		
ln Aβ1–42/<br>Aβ1–40<br>(LC–MS)	-0.035<br>1.000	**0.172**<br>**0.013**	**0.189**<br>**0.003**	**-0.303**<br>**<0.0001**	**0.238**<br>**<0.0001**	-0.037<br>1.000	**0.769**<br>**<0.0001**	**0.411**<br>**<0.0001**	
Composite<br>(LC–MS)	0.047<br>1.000	**-0.159**<br>**0.035**	**-0.183**<br>**0.005**	**0.329**<br>**<0.0001**	**-0.197**<br>**0.002**	0.085<br>1.000	**-0.629**<br>**<0.0001**	-0.006<br>1.000	**-0.863**<br>**<0.0001**

Supplementary table 3: Inter-biomarker correlations in dementia free-individuals with full blood biomarker and amyloid PET data (n = 441).

The Pearson correlation coefficient, r, and the Bonferroni-corrected *P* value for each correlation are shown, with $P < 0.05$ in bold.

Abbreviations: Aβ, amyloid-β; LC–MS, liquid chromatography–mass spectrometry; p-tau181, phospho-tau181.

	Value		
**Blood Biomarker**	Females, n = 223	Males, n = 218	*P*
Simoa plasma Aβ40, pg/mL	282 (255, 315)	297 (258, 327)	**0.025**
Simoa plasma Aβ42, pg/mL	19.1 (17.0, 21.8)	20.2 (16.5), 23.6	0.089
Simoa plasma Aβ42/Aβ40	0.067 (0.058, 0.076)	0.065 (0.057, 0.078)	0.886
Simoa plasma p-tau181, pg/mL	9.2 (6.5, 12.9)	9.2 (6.4, 12.9)	0.851
LC–MS plasma Aβ1–38, pg/mL	24.7 (21.6, 28.0)	24.8 (21.6, 28.0)	0.727
LC–MS plasma Aβ1–40, pg/mL	283 (254, 313)	286 (258, 314)	0.402
LC–MS plasma Aβ1–42, pg/mL	28.2 (23.4, 33.1)	29.1 (23.6, 33.6)	0.443
LC–MS plasma Aβ-3–40, pg/mL	29.8 (24.0, 35.1)	30.6 (24.6, 35.8)	0.693
LC–MS plasma Aβ1–42/Aβ1–40	0.098 (0.088, 0.111)	0.101 (0.084, 0.115)	0.453
LC–MS composite	0.030 (0.854)	-0.033 (0.738)	0.406

Supplementary table 4: Unadjusted differences in blood biomarkers by sex in dementia-free individuals, n=441.

Values are median (interquartile range) for skewed variables and mean (standard deviation) for LC–MS composite. Mann-Whitney U test p values are indicated for skewed variables, and t test p value is indicated for LC–MS composite.

Abbreviations: Aβ, amyloid-β; LC–MS, liquid chromatography–mass spectrometry; p-tau181, phospho-tau181.

	Model 1, n = 441		Model 2, n = 441		Model 3, n = 439		Model 4, n = 427		Model 5, n = 441	
**Blood Biomarker**	log-fold increase per year of age (95% CI)	*P*	log-fold increase per year of age (95% CI)	*P*	log-fold increase per year of age (95% CI)	*P*	log-fold increase per year of age (95% CI)	*P*	log-fold increase per year of age (95% CI)	*P*
Simoa Aβ40	0.007 (-0.016, 0.032)	0.523	0.007 (-0.017, 0.031)	0.582	0.010 (-0.015, 0.034)	0.435	0.007 (-0.017, 0.032)	0.558	0.006 (-0.017, 0.030)	0.603
Simoa Aβ42	0.065 (0.022, 0.107)	**0.001**	0.070 (0.028, 0.111)	**0.001**	0.074 (0.033, 0.115)	**0.001**	0.071 (0.029, 0.113)	**0.001**	0.069 (0.028, 0.110)	**0.001**
Simoa Aβ42/Aβ40	0.065 (0.022, 0.107)	**0.003**	0.063 (0.021, 0.105)	**0.004**	0.064 (0.022, 0.107)	**0.003**	0.064 (0.021, 0.107)	**0.004**	0.063 (0.021, 0.105)	**0.004**
Simoa plasma p-tau-181	0.382 (0.303, 0.461)	**<0.0001**	0.389 (0.313, 0.465)	**<0.0001**	0.380 (0.303, 0.457)	**<0.0001**	0.392 (0.315, 0.469)	**<0.0001**	0.388 (0.312, 0.464)	**<0.0001**
LC–MS Aβ1–38	-0.089 (-0.115,-0.063)	**<0.0001**	-0.090 (-0.116, -0.064)	**<0.0001**	-0.091 (-0.117, -0.064)	**<0.0001**	-0.089 (-0.116, -0.063)	**<0.0001**	-0.090 (-0.116, -0.064)	**<0.0001**
LC–MS Aβ1–40	-0.081 (-0.105, -0.056)	**<0.0001**	-0.082 (-0.107, -0.058)	**<0.0001**	-0.083 (-0.108, -0.058)	**<0.0001**	-0.081 (-0.106, -0.056)	**<0.0001**	-0.083 (-0.107, -0.058)	**<0.0001**
LC–MS Aβ1–42	-0.181 (-0.217, -0.144)	**<0.0001**	-0.185 (-0.219, -0.151)	**<0.0001**	-0.182 (-0.216, -0.147)	**<0.0001**	-0.184 (-0.219, -0.150)	**<0.0001**	-0.185 (-0.219, -0.152)	**<0.0001**
LC–MS Aβ3–40	-0.174 (-0.213, -0.135)	**<0.0001**	-0.174 (-0.213, -0.135)	**<0.0001**	-0.176 (-0.216, -0.137)	**<0.0001**	-0.181 (-0.220, -0.142)	**<0.0001**	-0.174 (-0.213,-0.136)	**<0.0001**
LC–MS Aβ1–42/Aβ1–40	-0.100 (-0.130, -0.069)	**<0.0001**	-0.103 (-0.131, -0.075)	**<0.0001**	-0.099 (-0.127, -0.071)	**<0.0001**	-0.103 (-0.132, -0.074)	**<0.0001**	-0.103 (-0.131, -0.075)	**<0.0001**
	Fold increase per year of age (95% CI)	*P*	Fold increase per year of age (95% CI)	*P*	Fold increase per year of age (95% CI)	*P*	Fold increase per year of age (95% CI)	*P*	Fold increase per year of age (95% CI)	*P*
LC–MS composite	0.201 (0.092, 0.309)	**0.0003**	0.214 (0.117, 0.311)	**<0.0001**	0.209 (0.112, 0.306)	**<0.0001**	0.202 (0.102, 0.302)	**0.0001**	0.214 (0.117, 0.311)	**0.0001**

Supplementary table 5: Associations of blood biomarkers with age in dementia-free individuals (n=441).

Linear regression coefficients for age are shown with their *P* values. Excepting LC–MS composite, log-transformed plasma amyloid outcomes were used.
Model 1: Plasma amyloid ~ age
Model 2: Plasma amyloid ~ age + sex + *APOE* *ε*4 carrier status + SUVR
Model 3: Plasma amyloid ~ age + sex + *APOE* *ε*4 carrier status + SUVR + total intracranial volume + whole brain volume
Model 4: Plasma amyloid ~ age + sex + *APOE* *ε*4 carrier status + SUVR + total intracranial volume + white matter hyperintensity volume
Model 5: Plasma amyloid ~ age + sex + *APOE* *ε*4 carrier status + SUVR + PACC

Abbreviations: Aβ, amyloid-β; *APOE* ε4, apolipoprotein E gene epsilon 4 allele; CI, confidence interval; LC–MS, liquid chromatography–mass spectrometry; PACC, pre-clinical Alzheimer's cognitive composite; p-tau181, phospho-tau181; SUVR, standardised uptake value ratio.

	Age		Male		*APOE ε4* carrier		SUVR		
**Blood Biomarker**	Log-fold change (95% CI)	*P*	Log-fold change (95% CI)	*P*	Log-fold change (95% CI)	*P*	Log-fold change (95% CI)	*P*	Adjusted R^2
Simoa plasma Aβ40	0.007 (-0.017, 0.031)	0.582	0.026 (-0.007, 0.058)	0.118	-0.023 (-0.061, 0.015)	0.233	0.080 (-0.154, 0.313)	0.502	0.001
Simoa plasma Aβ42	0.070 (0.028, 0.111)	**0.001**	0.035 (0.002, 0.069)	**0.037**	-0.019 (-0.057, 0.019)	0.324	0.048 (-0.186, 0.282)	0.687	0.012
Simoa plasma Aβ42/Aβ40	0.063 (0.021, 0.105)	**0.004**	-0.006 (-0.064, 0.051)	0.828	-0.034 (-0.101, 0.033)	0.320	-0.552 (-0.965, -0.140)	**0.009**	0.034
Simoa plasma p-tau-181	0.389 (0.313, 0.465)	**<0.0001**	-0.037 (-0.140, 0.066)	0.478	0.094 (-0.026, 0.214)	0.126	2.038 (1.297, 2.779)	**<0.0001**	0.237
LC–MS plasma Aβ1–38	-0.090 (-0.116, -0.064)	**<0.0001**	0.0001 (-0.036, 0.036)	0.997	-0.032 -(0.074, 0.009)	0.127	-0.002 (-0.258, 0.255)	0.990	0.090
LC–MS plasma Aβ1–40	-0.082 (-0.107, -0.058)	**<0.0001**	0.011 (-0.022, 0.045)	0.505	-0.042 (-0.081, -0.003)	**0.034**	0.002 (-0.238, 0.242)	0.988	0.090
LC–MS plasma Aβ1–42	-0.185 (-0.219, -0.151)	**<0.0001**	0.012 (-0.034, 0.058)	0.598	-0.086 (-0.140, -0.033)	**0.002**	-1.104 (-1.434, -0.774)	**<0.0001**	0.292
LC–MS plasma Aβ3–40	-0.174 (-0.213, -0.135)	**<0.0001**	0.002 (-0.052, 0.055)	0.955	-0.010 (-0.072, 0.052)	0.746	0.017 (-0.365, 0.400)	0.929	0.142
LC–MS plasma Aβ1–42/Aβ1–40	-0.103 (-0.131, -0.075)	**<0.0001**	0.001 (-0.037, 0.039)	0.958	-0.044 -0.089, -0.0001	**0.050**	-1.106 (-1.379, -0.833)	**<0.0001**	0.230
	Coefficient (95% CI)	*P*	Coefficient (95% CI)	*P*	Coefficient (95% CI)	*P*	Coefficient (95% CI)	*P*	Adjusted R^2
LC–MS plasma composite	0.214 (0.117, 0.311)	**<0.0001**	-0.035 (-0.167, 0.097)	0.605	0.225 (0.072, 0.379)	**0.004**	4.251 (3.304, 5.200)	**<0.0001**	0.224

Supplementary table 6: Associations of blood biomarkers with SUVR and APOE ε4 carrier status, adjusted for age and sex, in dementia-free individuals (n=441).

Log-fold change is shown for biomarkers that were log-transformed prior to linear regression (all except the LC–MS plasma composite, for which a linear regression coefficient is shown), with their p values and the overall R2 of each multivariate model.

Abbreviations: Aβ, amyloid-β; *APOE* ε4, apolipoprotein E gene epsilon 4 allele; CI, confidence interval; LC–MS, liquid chromatography–mass spectrometry; p-tau181, phospho-tau181; SUVR, standardised uptake value ratio.

	Biomarker alone		Biomarker + age + sex + *APOE* ε4 carrier status	
	AUC	95% CI	AUC	95% CI
Age + Sex + *APOE* ε4 carrier status	-	-	0.693	0.624, 0.763
Simoa plasma Aβ40	0.534	0.461, 0.607	0.717	0.655, 0.778
Simoa plasma Aβ42	0.570	0.493, 0.647	0.694	0.625, 0.764
Simoa plasma Aβ42/Aβ40	0.610	0.534, 0.685	0.720	0.655, 0.784
Simoa plasma p-tau181	0.720	0.657, 0.783	0.784	0.733, 0.834
LC–MS plasma Aβ1–38	0.503	0.430, 0.576	0.696	0.629, 0.763
LC–MS plasma Aβ1–40	0.494	0.421, 0.566	0.692	0.622, 0.763
LC–MS plasma Aβ1–42	0.734	0.680, 0.789	0.785	0.728, 0.842
LC–MS plasma Aβ3–40	0.508	0.435, 0.580	0.699	0.632, 0.765
LC–MS plasma Aβ1–42/Aβ1–40	0.817[a,b,d]	0.769, 0.866	0.839[f]	0.792, 0.886
LC–MS plasma composite	0.823[a,c,e]	0.776, 0.870	0.842[f]	0.797, 0.888

Supplementary table 7: Areas under the curve from ROC analyses of amyloid PET status incorporating blood biomarkers, with and without inclusion of age, sex and *APOE ε*4 carrier status, in cognitively normal individuals without prior neurological conditions (n=410).

DeLong tests:
[a] $P < 0.0001$ compared to Simoa plasma Aβ42/Aβ40
[b] $P = 0.008$ compared to Simoa plasma p-tau181
[c] $P = 0.005$ compared to Simoa plasma p-tau181
[d] $P = 0.005$ compared to age + sex + APOE ε4 carrier status
[e] $P = 0.002$ compared to age + sex + APOE ε4 carrier status
[f] $P < 0.0001$ compared to age + sex + APOE ε4 carrier status

Abbreviations: Aβ, amyloid-β; *APOE* ε4, apolipoprotein E gene epsilon 4 allele; CI, confidence interval; LC–MS, liquid chromatography–mass spectrometry; p-tau181, phospho-tau181.

	Biomarker alone		Biomarker + age + sex + *APOE* ε4 carrier status	
**Predictor(s)**	AUC	95% CI	AUC	95% CI
Individual Simoa biomarkers				
Age + sex + *APOE* ε4 carrier status	-	-	0.695	0.628, 0.762
Simoa plasma Aβ40	0.519	0.449, 0.588	0.713	0.653, 0.773
Simoa plasma Aβ42	0.590	0.516, 0.664	0.705	0.638, 0.771
Simoa plasma Aβ42/Aβ40	0.620	0.548, 0.691	0.727	0.665, 0.788
Simoa plasma p-tau181	0.707	0.646, 0.768	0.778	0.727, 0.828
Combinations of Simoa biomarkers				
Simoa plasma Aβ42/Aβ40 + p-tau181	0.696	0.631, 0.760	0.776	0.724, 0.828
Individual LC–MS biomarkers				
LC–MS plasma Aβ1–38	0.498	0.429, 0.568	0.697	0.632, 0.762
LC–MS plasma Aβ1–40	0.499	0.430, 0.568	0.694	0.626, 0.762
LC–MS plasma Aβ1–42	0.736	0.683, 0.788	0.789	0.735, 0.844
LC–MS plasma Aβ3–40	0.504	0.434, 0.574	0.699	0.633, 0.765
Combinations of LC–MS biomarkers				
LC–MS plasma Aβ1–42/Aβ1–40	0.817[a, c]	0.770, 0.864	0.841	0.796, 0.886
LC–MS plasma composite	0.820[b, c]	0.775, 0.866	0.843	0.798, 0.887
Combinations of LC–MS and Simoa biomarkers				
LC–MS plasma Aβ1–42/Aβ1–40 + Simoa plasma p-tau181	0.826	0.779, 0.872	0.851	0.807, 0.895
LC–MS plasma composite + Simoa plasma p-tau181	0.829	0.784, 0.874	0.850	0.808, 0.893

Supplementary table 8: Areas under the curve from ROC analyses of amyloid PET status incorporating combinations of plasma biomarkers, with and without inclusion of age, sex and *APOE* ε4 carrier status, in dementia-free individuals (n=441).

[a] P = 0.004 compared to Age + Sex + *APOE* ε4 carrier status; P = 0.002 compared to Simoa plasma p-tau181
[b] P = 0.002 compared Age + Sex + *APOE* ε4 carrier status; P = 0.001 compared to Simoa plasma p-tau181
[c] $P < 0.0001$ compared to Simoa plasma Aβ42/Aβ40

Abbreviations: Aβ, amyloid-β; *APOE* ε4, apolipoprotein E gene epsilon 4 allele; CI, confidence interval; LC–MS, liquid chromatography–mass spectrometry; p-tau181, phospho-tau181.

	All dementia-free individuals (n = 441)		*APOE* ε4 non-carriers (n = 315)		*APOE* ε4 carriers (n = 126)	
**Predictor(s)**	AUC	95% CI	AUC	95% CI	AUC	95% CI
Simoa plasma Aβ42/Aβ40	0.620	0.548, 0.691	0.642	0.532, 0.752	0.546	0.441, 0.651
Simoa plasma p-tau181	0.707	0.646, 0.768	0.731	0.644, 0.819	0.649	0.550, 0.749
LC–MS plasma Aβ1–42/Aβ1–40	0.817[a, c]	0.770, 0.864	0.816[d]	0.737, 0.895	0.757[g]	0.674, 0.840
LC–MS plasma composite	0.820[b, c]	0.775, 0.866	0.827[e, f]	0.754, 0.900	0.755[h]	0.672, 0.838

Supplementary table 9: Subgroup analysis: areas under the curve from ROC analyses of amyloid PET status incorporating individual biomarkers, in dementia-free APOE ε4 non-carriers (n = 315) and carriers (n = 126).

[a] $P = 0.002$ compared to Simoa plasma p-tau181
[b] $P = 0.001$ compared to Simoa plasma p-tau181
[c] $P < 0.0001$ compared to Simoa plasma Aβ42/Aβ40
[d] $P = 0.005$ compared to Simoa plasma Aβ42/Aβ40
[e] $P = 0.001$ compared to Simoa plasma Aβ42/Aβ40
[f] $P = 0.043$ compared to Simoa plasma p-tau181
[g] $P = 0.001$ compared to Simoa plasma Aβ42/Aβ40
[h] $P = 0.002$ compared to Simoa plasma Aβ42/Aβ40

Abbreviations: Aβ, amyloid-β; APOE ε4, apolipoprotein E gene epsilon 4 allele; CI, confidence interval; LC–MS, liquid chromatography–mass spectrometry; p-tau181, phospho-tau181.

SUVR cut-point	0.57		0.59		0.61		0.63		0.65	
Simoa Aβ42/Aβ40 cut-point	0.068		0.068		0.058		0.058		0.058	
Amyloid PET–positive (%)	29.9		22.5		18.6		15.9		12.9	
Amyloid status	PET–positive	PET–negative	PET–positive	PET–negative	PET–positive	PET–negative	PET–positive	PET–negative	PET–positive	PET–negative
Plasma–positive	92	145	70	167	36	79	34	81	30	85
Plasma-negative	40	164	29	175	46	280	36	290	27	399
Concordance, % of total	62.3		59.6		71.7		73.5		74.6	
Discordance, % of total	37.7		40.4		28.3		26.5		25.4	
Plasma–positive, PET–negative individuals, % of discordance	78.4		85.2		63.2		69.2		75.9	
Sensitivity %	71.2		72.7		45.1		48.6		52.6	
Specificity%	52.8		50.8		78.0		79.0		78.4	

Supplementary table 10: Influence of SUVR cut-point for amyloid PET status on Simoa Aβ42/Aβ40 performance in dementia-free individuals in Insight 46 (n=441).

Abbreviations: Aβ, amyloid-β; p-tau181, phospho-tau181; SUVR, standardised uptake value ratio.

**SUVR cut-point**	**0.57**		**0.59**		**0.61**		**0.63**		**0.65**	
Amyloid PET–positive (%)	29.9		22.5		18.6		15.9		12.9	
Amyloid status	PET–positive	PET–negative	PET–positive	PET–negative	PET–positive	PET–negative	PET–positive	PET–negative	PET–positive	PET–negative
Plasma–positive	80	93	64	109	58	115	50	123	41	132
Plasma-negative	52	216	35	233	24	244	20	248	16	252
Concordance, % of total	67.1		67.3		68.5		67.6		66.4	
Discordance, % of total	32.9		32.7		31.5		32.4		33.6	
Plasma–positive, PET–negative individuals, % of discordance	64.1		75.7		82.7		86.0		89.2	
Sensitivity %	60.6		64.6		70.7		71.4		71.9	
Specificity %	70.2		68.4		68.2		67.1		65.9	

Supplementary table 11: Influence of SUVR cut-point for amyloid PET status on Simoa p-tau181 performance in dementia-free individuals in Insight 46 (n=441).

Across this range of SUVR cut-points, the Youden’s index cut-point for Simoa p-tau181 (used to assign plasma status) remained constant at 10.8 pg/mL.

Abbreviations: Aβ, amyloid-β; p-tau181, phospho-tau181; SUVR, standardised uptake value ratio.

SUVR cut-point	0.57		0.59		0.61		0.63		0.65	
Amyloid PET–positive (%)	29.9		22.5		18.6		15.9		12.9	
Amyloid status	PET–positive	PET–negative	PET–positive	PET–negative	PET–positive	PET–negative	PET–positive	PET–negative	PET–positive	PET–negative
Plasma–positive	96	79	80	95	71	104	63	112	53	122
Plasma-negative	36	230	19	247	11	255	7	259	4	262
Concordance, % of total	73.9		74.1		73.9		73.0		71.4	
Discordance, % of total	26.1		25.9		26.1		27.0		28.6	
Plasma–positive, PET–negative individuals, % of discordance	68.7		83.3		90.4		94.1		96.8	
Sensitivity %	72.7		80.8		86.6		90.0		94.7	
Specificity%	74.1		73.1		71.9		70.6		67.5	

Supplementary table 12: Influence of SUVR cut-point for amyloid PET status on LC–MS Aβ1–42/1–40 performance in dementia-free individuals in Insight 46 (n=441).

Across this range of SUVR cut-points, the Youden's index cut-point for LC–MS Aβ1–42/1–40 (used to assign plasma status) remained constant at 0.095.

Abbreviations: Aβ, amyloid-β; LC–MS, liquid chromatography–mass spectrometry; p-tau181, phospho-tau181; SUVR, standardised uptake value ratio.

**SUVR cut-point**	**0.57**		**0.59**		**0.61**		**0.63**		**0.65**	
LC–MS composite cut-point	-0.076		-0.076		-0.049		-0.049		-0.049	
Amyloid PET–positive (%)	29.9		22.5		18.6		15.9		12.9	
Amyloid status	PET–positive	PET–negative	PET–positive	PET–negative	PET–positive	PET–negative	PET–positive	PET–negative	PET–positive	PET–negative
Plasma–positive	102	100	83	119	75	123	67	131	54	144
Plasma-negative	30	209	16	223	7	236	3	240	3	240
Concordance, % of total	70.5		69.4		70.1		69.6		66.7	
Discordance, % of total	29.5		30.6		29.5		30.4		33.3	
Plasma–positive, PET–negative individuals, % of discordance	76.9		88.1		94.6		97.8		98.0	
Sensitivity %	77.3		83.8		91.5		95.7		94.7	
Specificity%	67.6		64.9		65.7		64.7		62.5	

Supplementary table 13: Influence of SUVR cut-point for amyloid PET status on LC–MS composite performance in dementia-free individuals in Insight 46 (n=445).

Abbreviations: LC–MS, liquid chromatography–mass spectrometry; p-tau181, phospho-tau181; SUVR, standardised uptake value ratio.

Supplementary References

Andreasson U, Perret-Liaudet A, van Waalwijk van Doorn LJ, Blennow K, Chiasserini D, Engelborghs S, *et al.* A Practical Guide to Immunoassay Method Validation. Front Neurol 2015; 6: 179.
